# Hydrogenation of unactivated enamines to tertiary amines: rhodium complexes of fluorinated phosphines give marked improvements in catalytic activity

**DOI:** 10.3762/bjoc.11.70

**Published:** 2015-05-05

**Authors:** Sergey Tin, Tamara Fanjul, Matthew L Clarke

**Affiliations:** 1 School of Chemistry, University of St Andrews, EaStCHEM, St Andrews, KY16 9ST, Fife, UK, FAX +44 1334 463808; 2 Chirotech Technology Centre, Dr. Reddy’s Laboratories, Unit 410 Cambridge Science Park, Milton Road, Cambridge, CB4 0PE, UK

**Keywords:** alkenes, homogeneous catalysis, hydrogenation, renewable solvents, tertiary amines

## Abstract

In the hydrogenation of sluggish unactivated enamine substrates, Rh complexes of electron-deficient phosphines are demonstrated to be far more reactive catalysts than those derived from triphenylphosphine. These operate at low catalyst loadings (down to 0.01 mol %) and are able to reduce tetrasubstituted enamines. The use of the sustainable and environmentally benign solvent (*R*)-limonene for the reaction is also reported with the amine isolated by acid extraction.

## Introduction

A potentially very direct method to produce tertiary amines is by the hydrogenation of enamines. While the hydrogenations of enamides, bearing coordinating acyl substituents is probably the most developed and studied of all hydrogenation processes, studies on the hydrogenation of unactivated enamines are scarce and several important problems need to be solved. Some time ago, the enantioselective variant was highlighted by several pharma companies as one of the more important aspirational transformations for production of pharmaceuticals [1–3]. Several examples of highly enantioselective and quite reactive processes have appeared for enamines that are activated by a chelating group, or can potentially isomerise to an NH imine during catalysis [4–5]. A few papers have appeared with good enantioselectivity for some quite specific enamines, but despite the importance of these contributions, catalyst loadings around 1 mol % are used [6–13]. Commercial applications generally require catalyst loading below 0.05 mol %. We are not aware of any achiral or chiral homogeneous catalysts that promote these reactions at this substrate/catalyst ratio, so the intrinsic lower reactivity of these substrates needs to be addressed with new catalysts. In a recent study on hydroaminomethylation, i.e., domino hydroformylation–enamine formation–enamine hydrogenation, we noted that the enamine hydrogenation was the slowest reaction in the process, and use of an electron-deficient phosphine sped up the reduction step significantly [14]. DFT calculations revealed that in the hydrogenation of these aldehyde-derived enamines, the final stage of hydrogenation, reductive elimination was the rate-determining step. This is in contrast to nearly all studies on homogeneous hydrogenation of alkenes where oxidative addition, and probably more often migratory insertions are rate-determining and accelerated by electron-rich phosphine ligands. Prior to embarking on a quest for highly active Rh catalysts for enantioselective enamine hydrogenation, we investigated if more commonly encountered enamine substrates are also reduced much faster using Rh complexes of electron-withdrawing phosphines. In this paper, we report how a range of enamines can be successfully hydrogenated in high yield using low levels of rhodium, including some very deactivated enamines that do not hydrogenate using conventional catalysts.

## Results and Discussion

**Scheme 1:**
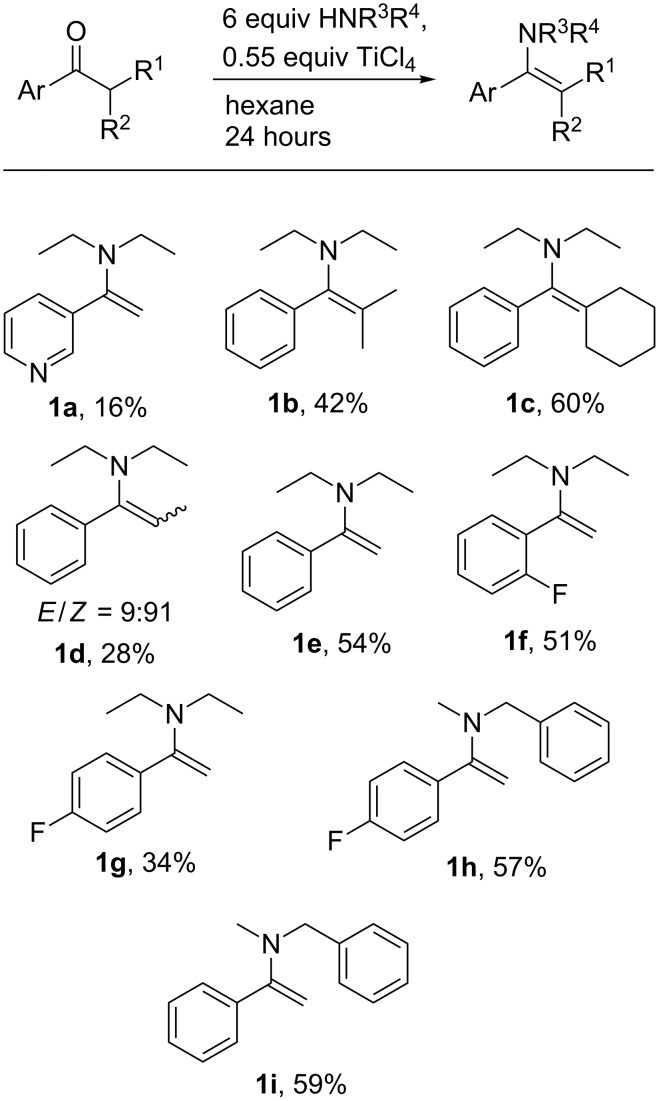
Synthesis of enamines from ketones with percentage yields.

The majority of the enamines produced in this study were synthesised from the parent ketones and secondary amines by adapting literature procedures (Scheme 1) [15].

Since isolation of pure enamines is not a completely trivial task, Supporting Information File 1 gives full details for the synthesis and purification of enamines **1a**–**i**. One of the main modifications made to the synthetic procedure is at the end of the reaction, wet diethyl ether was added in order to precipitate all titanium salts (this strategy was previously used after formation of imine bonds using TiCl_4_) [16]. Enamine **1g** was more stable than all other enamines with disubstituted double bond studied here; no hydrolysis was observed in wet chloroform even after 6 hours. It is also worth mentioning that tetrasubstituted enamines are very stable towards hydrolysis. Consequently, enamines **1b** and **1c** were isolated by acid-basic work-up with purities of over 99% (see Supporting Information File 1 for details).

**Scheme 2:**
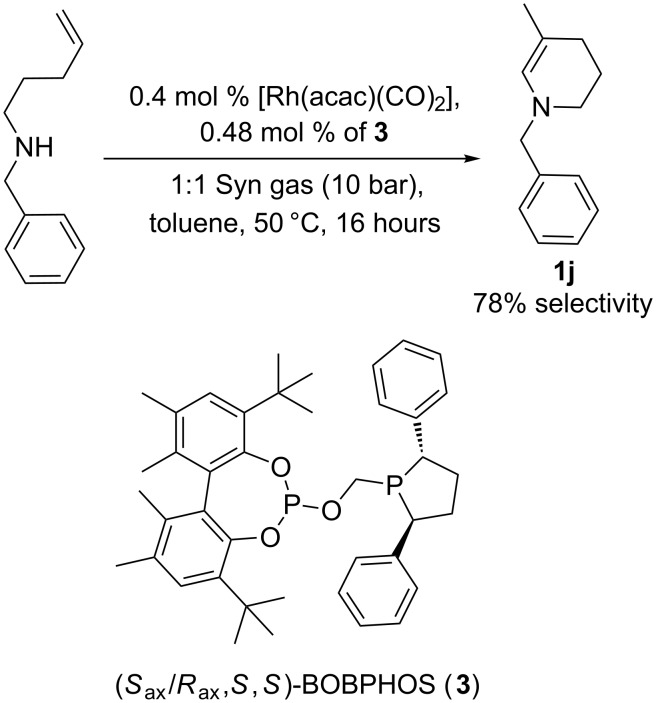
Branched-selective intramolecular hydroaminovinylation (60% isolated yield of **1j**).

Enamine **1j** cannot be prepared using this strategy, and therefore we developed a new branched-selective hydroaminovinylation procedure [17–20]. Some time ago, this enamine was detected in a product mixture with up to 39% selectivity [20]. A key aspect that prevents better selectivity is that, in general, Rh catalysed hydroformylations of ‘alkyl’ alkenes of type RCH_2_CH=CH_2_ give mainly the linear product [21–22]. Since we had recently discovered that Rh complexes of the ‘BOBPHOS’ ligand unexpectedly give unprecedented branched regioselectivity in enantioselective hydroformylation of alkyl- and arylalkenes [23–24], we reconsidered this cyclisation reaction using the new catalyst (Scheme 2). We were pleased to find that the selectivity is increased to 78%. Since the desired product is achiral, there is no need to use enantiopure BOBPHOS for this synthesis. When the reaction was performed on 8 mmol scale, using a BOBPHOS sample made from racemic biphenol derivative **3**, the enamine **1j** was isolated in an overall yield of 60%.

**Scheme 3:**
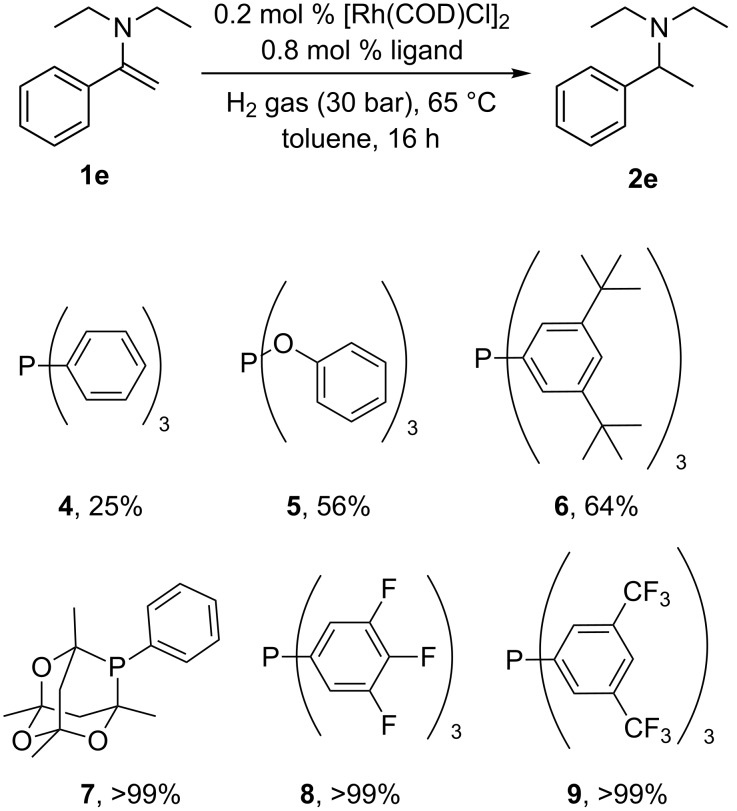
Conversion of **1e** to **2e** using ligands **4–9**.

We initially wanted to establish the generality of the previous observation that electron-withdrawing ligands enhance the rate of Rh catalysed hydrogenation relative to more electron donating ligands such as triphenylphosphine. The hydrogenation of enamine **1e** at a S/Rh ratio of 250 at 65 °C proceeded at a suitable rate, such that simply measuring conversion at the times given provides a meaningful measure of the relative rates of hydrogenation for Rh catalysts derived from electron-donating and electron-withdrawing ligands. A screen of monodentate ligands was performed for hydrogenation of **1e** with catalysts derived from ligands **4**–**9** (Scheme 3).

Compared to triphenylphosphine, more electron-poor ligands, particularly commercially available **7**, **8** [25] or also commercial product **9**, show faster rates of hydrogenation of **1e**. It can be envisaged that other less electron-donating phosphines could also be used to good effect, providing they are stable under the reaction conditions. It is possible that stability is an issue with the strong π-acceptor ligand triphenylphosphite. We note here that an earlier attempt by some of us using chiral phosphites in this type of reaction gave very low conversions to product under these conditions. The ligand electronic effect clearly supports our earlier proposal of the reductive elimination as rate determining step in this process [14]. Using readily available and simple ligand **8**, combined with [Rh(COD)Cl]_2_, we also studied the hydrogenation of a range of other enamines with [Rh(COD)Cl]_2_/PPh_3_ as a control. Table 1 shows very clearly the improved performance of the less strongly donating phosphine ligand for this process. For enamines **1g**, **1h** and **1i**, experiments with much lower catalyst loadings were performed in order to prove that the rate of hydrogenation is faster when **8** is used instead of **4**. Of particular note is the hydrogenation of the deactivated enamines **1b** and **1c**. It is well known that, even without deactivating nitrogen substituents, the hydrogenation of tetrasubstituted alkenes is not generally achieved with Rh catalysts [26–27]; Crabtree’s catalyst is often used to accomplish this type of task [26–27]. The ability of this catalyst combination to conduct this type of transformation, as shown in Table 1, entries 6 and 8 are of synthetic value.

**Table 1:** Hydrogenation of enamines with Rh catalysts of PPh_3_ vs ligand **8**.

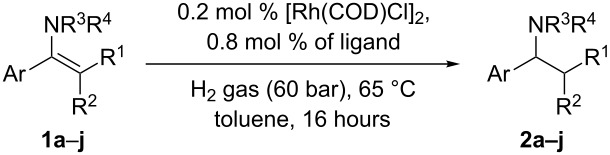				

Entry^a^	Enamine	Ligand	Time, h	Amine, %^b^

1	**1a**	**4**	16	<1
2		**8**	16	1

3	**1b**	**4**	16	2
4		**8**	16	77
5		**8**	24	90
6^c^		**8**	24	>99

7	**1c**	**4**	24	<1
8		**8**	24	67

9	**1d**	**4**	16	6
10		**8**	16	>99
11^d^		**8**	16	10

12	**1e**	**4**	16	35
13		**8**	16	>99

14	**1f**	**4**	16	57
15		**8**	16	>99

16	**1g**	**4**	16	98
17		**8**	16	>99
18^e^		**4**	16	5
19^e^		**8**	16	23

20	**1h**	**4**	16	>99
21		**8**	16	>99
22^e^		**4**	16	14
23^e^		**8**	16	64

24	**1i**	**4**	16	>99
25		**8**	16	>99
26^e^		**4**	16	15
27^e^		**8**	16	70
28^f^		**8**	90	83
29^f^		**9**	66	91
30^g^		**8**	16	>99

31	**1j**	**4**	16	54
32		**8**	16	>99

^a^General conditions: 1 mmol of enamine, 0.2 mol % of [Rh(COD)Cl]_2_, 0.8 mol % ligand, 0.1 mL of 1-methylnaphthalene as an internal standard, 60 bar of H_2_ gas, toluene as a solvent. ^b^Determined by ^1^H NMR relative to 1-methylnaphthalene. ^c^Catalyst loading doubled. ^d^30 equivalents of pyridine relative to Rh were added. ^e^0.025 mol % of [Rh(COD)Cl]_2_, 0.1 mol % of ligand. ^f^0.01 mol % of [Rh(COD)Cl]_2_, 0.04 mol % of ligand; scale is 10.0 mmol of enamine. ^g^Pressure of H_2_ = 5 bar; scale is 2.0 mmol of enamine.

Trisubstituted enamine **1d** (Table 1, entry 9) is slower to reduce than all disubstituted enamines (except entry 1). Enamine **1j** (Table 1, entry 31) shows a much faster rate of hydrogenation than **1d** (entry 9), presumably due to the fact that there is a ring strain due to the double bond in a 6-membered ring, which is released after the double bond is hydrogenated. **1a** does not get hydrogenated with the catalysts studied. It is likely that this is due to the substrate binding to the catalyst via the pyridine nitrogen and deactivating the catalyst. In order to provide support for this, the normally high-yielding hydrogenation of **1d** was carried out in the presence of 30 equivalents of pyridine relative to Rh, and the conversion dropped to 10%. Comparing Table 1, entries 12, 14 and 16, it is clear that electron-poor enamines get hydrogenated faster. A possible reason for the enamine **1f** being reduced slower than **1g** may come from the fact that **1f** is a much more stable enamine (see enamine synthesis section, Supporting Information File 1).

It is well known that the reductive elimination is sped up with more bulky ligands – i.e., when the bulk around the transition state is larger, this step occurs more readily. Enamines **1h** and **1i** are more bulky due to their *N*-benzyl substituents, and therefore are hydrogenated with faster rates. Table 1, entry 29 represents a TON of 4550 mol mol^−1^ which is, to the best of our knowledge, the highest TON in an enamine hydrogenation reported up to date. We found it convenient to carry out these reactions at 30–60 bar of H_2_ gas (in order to compare reactivities of enamines with triphenylphosphine as a ligand). Full conversion is also possible at 5 bar pressure (Table 1, entry 30), but we did not observe product using a balloon of hydrogen (~1 bar). The latter observation contrasts somewhat with the results of reference [7], when using 1 mol % of a [Rh(diphosphine)(COD)] cation on a disubstituted enamine: complete conversion can be realised in 2–18 hours. It can be assumed that the catalysts used here are less efficient at activating hydrogen relative to more electron-rich metal systems, meaning below a certain pressure threshold, hydrogen activation does not proceed at a sufficient rate.

In order to prove that toluene is not the only solvent where an electronic effect holds, a polar protic solvent (MeOH) was chosen (Table 2). The electronic effect still holds in MeOH as a solvent, although it is less pronounced, and the best rate of conversion is found in toluene.

**Table 2:** Hydrogenation of enamine **1e** in toluene and methanol as solvents.

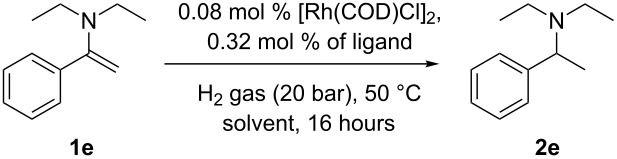			

Entry^a^	Solvent	Ligand	Amine, %^b^

1	toluene	**4**	4
2	toluene	**8**	93
3	methanol	**4**	43
4	methanol	**8**	57

^a^General conditions: 5.0 mmol of **1e**, 0.08 mol % of [Rh(COD)Cl]_2_, 0.32 mol % ligand, 0.5 mL of 1-methylnaphthalene as an internal standard, 20 bar of H_2_ gas, solvent. ^b^Determined by ^1^H NMR relative to 1-methylnaphthalene.

Another solvent explored in this study was (*R*)-limonene. This solvent is now being used as a green alternative to hexane in the cleaning industry and extraction [28–30], but barely has been exploited in synthetic chemistry so far [29]. While being an environmentally benign, fairly cheap, waste-derived chemical, it might seem counter-intuitive to use it in hydrogenation since it contains 2 double bonds itself. However, in hydrogenation of enamines, as was shown above, enamine hydrogenation benefits from electron-poor ligands, so the hope was that the enamine hydrogenation would be competitive over limonene hydrogenation. In addition, this solvent enables us to study the relative reactivities of these C=C bonds, as well as possibly giving a greener procedure. Examples of hydrogenation of **1h** in limonene as a solvent are shown in Table 3.

**Table 3:** Hydrogenation of enamine **1h** in (*R*)-limonene as a solvent.

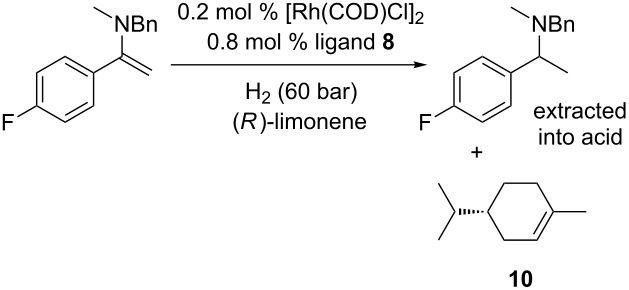					

Entry^a^	Ligand	Time, h	*T*, °C	Amine, %^b^	**10**, %^b^

1	**4**	16	40	0	48
2	**8**	16	40	74	20
3	**4**	20	45	11	65
4	**8**	20	45	92	23

^a^General conditions: 1.5 mmol of **1h**, 0.2 mol % of [Rh(COD)Cl]_2_, 0.8 mol % ligand, 0.15 mL of 1-methylnaphthalene as an internal standard, 60 bar of H_2_ gas, (*R*)-limonene. Ratio of solvent/enamine = 6.68:1. ^b^Determined by ^1^H NMR relative to 1-methylnaphthalene.

The results shown in Table 3 suggest that limonene is a promising solvent for this process. As expected, triphenylphosphine shows higher selectivity in hydrogenation of the limonene’s disubstituted double bond, and low conversion to amine. Ligand **8** allows good conversion to amine with relatively low amounts of limonene hydrogenated at the least substituted double bond. While this solvent is not completely inert, it is envisaged that the mixture of limonene and dihydrolimonene (**10**) would be a perfectly suitable solvent mixture to recycle and reuse. (*R*)-Limonene is a high boiling solvent, creating a disadvantage for processes where the solvents are removed by evaporation. However, in amine synthesis in general, amines are isolated by extraction into acid, and this was demonstrated here (see Supporting Information File 1). We suggest that (*R*)-limonene is worth considering as a sustainable, benign solvent for amine synthesis in the future.

## Conclusion

Overall, the primary outcome from this study is to demonstrate that highly active Rh catalysts for enamine hydrogenation are a possibility, but they require quite different ligands to those needed for enamide hydrogenation. From a synthetic perspective, large scale reduction processes generally prefer the use of hydrogen gas to any other reductant, since it potentially saves on cost, waste, atom-economy, solvent and water use; the catalysts identified here could be useful in this regard. While it is possible some heterogeneous hydrogenation catalysts could accomplish enamine reductions, the issues with functional group tolerance would be problematic in many cases. From a more general synthetic viewpoint, the use of reagents such as sodium triacetoxyborohydride or sodium cyanoborohydride can be appealing at small scale where the practical issues noted above are not so important. However, the formation of tertiary amines from aryl ketones using hydride reagents has been reported to be problematic [31]. In addition, the hydride reductions, whether carried out as reductive amination or reduction of enamines need stoichiometric acetic acid to promote the formation of the iminium ion that is the substrate reduced in hydride reductions, which might not be compatible with other functional groups. To the best of our knowledge, the hydrogenation of tetrasubstituted enamines has not been carried out before. The use of green, non-toxic and renewable solvent (*R*)-limonene is introduced here as a potentially promising solvent for amine synthesis. This solvent could prove a particularly useful green solvent for any reaction that involved an aqueous/organic work-up as purification step, particularly if catalysts could be recycled, although that is likely to be challenging in moisture sensitive catalytic hydrogenation chemistry.

The ligand electronic effects seem counter intuitive at first glance, but they support the finding by DFT calculations that enamine hydrogenation has a different rate determining step to most other alkene hydrogenations, and show that these observations are a general phenomenon of synthetic use, since some of the enamines studied here are rather unreactive using normal catalysts and/or in reductions using hydride reagents. Research on the creation of enantioselective enamine hydrogenation catalysts that can operate at industrially acceptable catalysts loadings may well benefit from chiral π-acceptor phosphines as ligands and this is being actively researched in our laboratory.

## Supporting Information

File 1 Full details of substrate syntheses, product isolation and characterisation, along with NMR spectra of substrates and products.
