# Supplementary material for: Hydrogenation of unactivated enamines to tertiary amines: rhodium complexes of fluorinated phosphines give marked improvements in catalytic activity
Source: Beilstein J Org Chem. 2015 May 5;11:622–7. doi: 10.3762/bjoc.11.70 (PMC4464316; doi:10.3762/bjoc.11.70)
Supplement: File 1 — Full details of substrate syntheses, product isolation and characterisation, along with NMR spectra of substrates and products. [file Beilstein_J_Org_Chem-11-622-s001.pdf]

Supporting Information

for

Hydrogenation of unactivated enamines to tertiary amines: rhodium complexes of fluorinated phosphines give marked improvements in catalytic activity

Sergey Tin[1], Tamara Fanjul[2] and Matthew L. Clarke[1]*

Address: [1]School of Chemistry, University of St Andrews, EaStCHEM, St Andrews, KY16 9ST, Fife, UK, FAX +44 1334 463808 and [2]Chirotech Technology Centre, Dr. Reddy's Laboratories, Unit 410 Cambridge Science Park, Milton Road, Cambridge, CB4 0PE, UK

Email: Matthew L. Clarke - mc28@st-andrews.ac.uk

*Corresponding author

Full details of substrate syntheses, product isolation and characterisation, along with NMR spectra of substrates and products

General experimental techniques

All hydroaminovinylation / hydrogenation procedures were carried out under inert conditions using standard schlenk techniques, and all solvents used were dried and degassed. Synthetic procedures of enamines using $TiCl_4$ were carried out in dry solvents under a nitrogen atmosphere. Work-ups of these reactions, as well as isolation of amines, were carried out in air. All materials were used as received, unless otherwise stated.

$[Rh(COD)Cl]_2$ was prepared according to the literature procedure.[1] Ligands **7**[2] and **8**[3] were made by the literature procedures. Ligand **3** was prepared by the literature procedure using a

racemic mixture of the aromatic diol.[4] Other materials were purchased from Sigma-Aldrich or Acros and were used without further purification.

^{1}H; ^{13}C; ^{19}F; ^{1}H,^{1}H COSY; ^{1}H,^{13}C HSQC and ^{1}H,^{13}C HMBC NMRs were acquired on Bruker Avance 500 (500 MHz), Bruker Avance 400 (400 MHz) or Bruker 300 (300 MHz).

Mass spectrometry and high-resolution mass spectrometry were carried out by Mrs Caroline Horsburgh at the University of St Andrews.

Infrared spectra (ν_{max}) were recorded on Shimadzu IRAffinity-1 using a Pike attenuated total reflectance (ATR) accessory. Only characteristic absorbances were assigned.

Elemental analyses were acquired by Mr. Stephen Boyer from London Metropolitan University.

Synthetic procedures.

Synthesis of *N*-benzylpent-4-en-1-amine (prepared according to the literature procedure).[5]

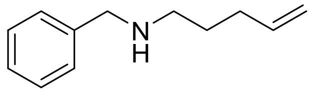

An ethanol solution (50 mL) of benzylamine (10.9 mL, 100 mmol), bromopentane (2.4 mL, 20.3 mmol) and sodium iodide (0.151 g, 1.01 mmol) was heated under reflux for 16 hours. After this time, the solvent was removed under the reduced pressure and dissolved in DCM (100 mL). The solution was washed with aqueous KOH (1 M, 50 mL), dried over KOH, filtered and solvent was removed under the reduced pressure. The product was purified by distillation under reduced pressure (90 °C, 1.5 mbar) to afford the desired product (2.036 g, 11.6 mmol, 57%) as a colourless liquid.

^{1}H NMR (300 MHz, $CDCl_3$, 298 K): δ_H = 7.27 – 7.09 (5H, m, Ar-$\underline{H}$), 5.72 (1H, ddt, $^{3}J_{HH}$ = 17.0 Hz, $^{3}J_{HH}$ = 10.3 Hz, $^{3}J_{HH}$ = 6.7 Hz, C$\underline{H}$), 4.98 – 4.62 (2H, m, =C$\underline{H_2}$), 3.68 (2H, s, Ar-C$\underline{H_2}$), 2.55 (2H, t, $^{3}J_{HH}$ = 7.3 Hz, N-C$\underline{H_2}$), 2.06 – 1.95 (2H, m, CH-C$\underline{H_2}$-), 1.51 (2H, qu, $^{3}J_{HH}$ = 7.5 Hz, C$\underline{H_2}$), 1.16 (1H, br s, N-$\underline{H}$).

^{13}C{^{1}H} NMR (75 MHz, $CDCl_3$, 298 K): δ_C = 141.1 (s, $^{Ar}\underline{C}$), 139.0 (s, $\underline{C}$H), 128.8 (s, $^{Ar}\underline{C}$H), 128.5 (s, $^{Ar}\underline{C}$H), 127.3 (s, $^{Ar}\underline{C}$H), 115.1 (s, =$\underline{C}H_2$), 54.5 (s, Ar-$\underline{C}H_2$), 49.4 (s, N-$\underline{C}H_2$), 32.0 (s, CH-$\underline{C}H_2$-), 29.8 (s, $\underline{C}H_2$).

MS (ES^+) *m/z*: 176.12 ($[MH]^+$, 100%).

Enamine synthesis (prepared according to the literature procedure).[6]

N,*N*-Diethyl-1-(pyridin-3-yl)ethenamine (**1a**)[7]

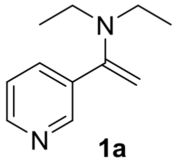

1a

A hexane solution (100 mL) of 1-(pyridin-3-yl)ethan-1-one (4.0 mL, 36.4 mmol) and diethylamine (22.7 mL, 218 mmol) was cooled to 0 °C and $TiCl_4$ (2.2 mL, 20.1 mmol) was added dropwise and the resulting suspension was left to stir for further 30 minutes followed by stirring the reaction mixture at room temperature for further 24 hours. After this time, the flask was opened to air, the solution was filtered and volatiles were removed in vacuo.

The product was purified by Kugelrohr distillation (oven T = 95 °C, 1.5 mbar) to afford the desired enamine (1.002 g, 5.68 mmol, 16%) as a pale yellow liquid.

^{1}H NMR (300 MHz, C_6D_6, 298 K): δ_H = 8.58 (1H, d, $^4J_{HH}$ = 2.2 Hz, N-^{Ar}C<u>H</u>-^{Ar}C), 8.43 (1H, dd, $^3J_{HH}$ = 4.9 Hz, $^4J_{HH}$ = 1.7 Hz, N-^{Ar}C<u>H</u>-CH), 7.62 (1H, dt, $^3J_{HH}$ = 7.8 Hz, $^4J_{HH}$ = 2.1 Hz, ^{Ar}C<u>H</u>-^{Ar}C), 7.14 (1H, dd, $^3J_{HH}$ = 8.0 Hz, $^4J_{HH}$ = 4.8 Hz, ^{Ar}C<u>H</u>), 4.10 (1H, s, one of C=C<u>H</u>$_2$), 4.06 (1H, s, one of C=C<u>H</u>$_2$), 2.88 (4H, q, $^3J_{HH}$ = 7.1 Hz, 2 x C<u>H</u>$_2$-CH_3), 0.93 (6H, t, $^3J_{HH}$ = 7.1 Hz, 2 x C<u>H</u>$_3$).

^{13}C NMR (75 MHz, $CDCl_3$, 298 K): δ_C = 152.0 (s, N-<u>C</u>), 149.5 (s, N-^{Ar}<u>C</u>H-^{Ar}C), 149.2 (s, N-^{Ar}<u>C</u>H), 136.8 (s, ^{Ar}<u>C</u>), 135.2 (s, ^{Ar}C-^{Ar}<u>C</u>H), 123.2 (s, ^{Ar}<u>C</u>H), 92.5 (s, C=<u>C</u>H$_2$), 45.4 (s, N-<u>C</u>H$_2$), 11.9 (s, <u>C</u>H$_3$).

MS (ES$^+$) *m/z*: 177.11 ([MH]$^+$, 100%), 149.12 ([M – C_2H_5]$^+$, 15).

(**1b**)

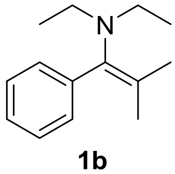

1b

A hexane solution (100 mL) of 2-methyl-1-phenylpropan-1-one (5.0 mL, 33.3 mmol) and diethylamine (21.0 mL, 202 mmol) was cooled to 0 °C and $TiCl_4$ (2.0 mL, 18.2 mmol) was added dropwise and the resulting suspension was left to stir for further 30 minutes followed by stirring the reaction mixture at room temperature for further 24 hours. After this time, the flask was opened to air and water-saturated diethyl ether (20 mL) was added. The solution

was filtered and volatiles were removed in vacuo. Crude enamine was dissolved in toluene (150 mL), exctacted into aqueous HCl (1 M, 3 × 100 mL) and the combined acidic fractions were basified with aqueous NaOH (1 M) until pH 10–12. The enamine was then extracted into ethyl acetate (3 × 70 mL), the combined organic fractions were washed with water (50 mL) and brine (50 mL), dried over $MgSO_4$ and solvent was removed under reduced pressure. The enamine was dried in vacuo for 2 hours to afford the desired product (2.848 g, 14.0 mmol, 42%) as a pale yellow liquid.

Due to high stability of this enamine, it was found that acid-base work-up is a suitable method of purifying enamines with tetrasubstituted double bond studied in this paper (another example is enamine **1c**).

^{1}H NMR (400 MHz, C_6D_6, 298 K): δ_H = 7.23 – 7.17 (2H, m, Ar-H), 7.16 – 7.07 (3H, m, Ar-H), 2.61 (4H, q, $^{3}J_{HH}$ = 7.1 Hz, 2 x CH_2), 1.96 (3H, s, C-CH_3), 1.58 (3H, s, C-CH_3), 1.03 (6H, t, $^{3}J_{HH}$ = 7.1 Hz, 2 x CH_2-CH_3) .

^{13}C{^{1}H} NMR (101 MHz, $CDCl_3$, 298 K): δ_C = 140.9 (s, N-C), 138.1 (s, ^{Ar}C), 130.1 (s, ^{Ar}CH), 127.5 (s, ^{Ar}CH), 126.7 (s, C-(CH_3)$_2$), 126.3 (s, ^{Ar}CH), 47.1 (s, N-CH_2), 21.0 (s, C-CH_3), 20.0 (s, C-CH_3), 14.2 (s, CH_2-CH_3).

MS (ES$^+$) *m/z*: 204.17 ([MH]$^+$, 100%); Found(ES$^+$) 204.1742 ([MH]$^+$), $C_{14}H_{22}N^+$ requires 204.1747.

IR (neat) *v*, cm^{-1}: 2968, 2820, 1489, 1441, 1375, 1219, 1109, 1072.

(1c)

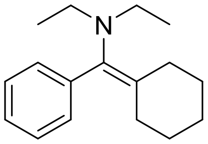

1c

A hexane solution (90 mL) of cyclohexyl(phenyl)methanone (4.911 g, 26.1 mmol) and diethylamine (16.5 mL, 159 mmol) was cooled to 0 °C and $TiCl_4$ (1.6 mL, 14.6 mmol) was added dropwise and the resulting suspension was left to stir for further 30 minutes followed by stirring the reaction mixture at room temperature for further 24 hours. After this time, the flask was opened to air and water-saturated diethyl ether (16 mL) was added. The solution

was filtered and volatiles were removed in vacuo. Crude enamine was dissolved in toluene (120 mL), exctacted into aqueous HCl (1 M, 3 × 80 mL) and the combined acidic fractions were basified with aqueous NaOH (1 M) until pH 10–12. The enamine was then extracted into ethyl acetate (3 × 50 mL), the combined organic fractions were washed with water (40 mL) and brine (40 mL), dried over $MgSO_4$ and solvent was removed under reduced pressure. The enamine was dried in vacuo for 2 hours to afford the desired product (3.811 g, 15.7 mmol, 60%) as viscous pale yellow oil.

Anal. Calcd. for $C_{17}H_{25}N$ (found): C, 83.89 (83.74); H, 10.35 (10.28); N, 5.75 (5.96).

^{1}H NMR (400 MHz, C_6D_6, 298 K): δ_H = 7.24 – 7.08 (5H, m, Ar-H), 2.64 – 2.57 (6H, m, 2 x N-CH_2 and one -CH_2), 2.07 – 2.02 (2H, m, CH_2), 1.65 – 1.57 (2H, m, CH_2), 1.53 – 1.46 (2H, m, CH_2), 1.45 – 1.38 (2H, m, CH_2), 1.05 (6H, t, $^{3}J_{HH}$ = 7.1 Hz, 2 x CH_3).

^{13}C NMR (101 MHz, $CDCl_3$, 298 K): δ_C = 137.8 (C), 137.6 (C), 135.5 (C), 129.9 (s, ^{Ar}CH), 127.5 (s, ^{Ar}CH), 126.3 (s, ^{Ar}CH), 47.1 (s, N-CH_2), 31.5 (s, CH_2), 29.7 (s, CH_2), 28.6 (s, CH_2), 27.9 (s, CH_2), 27.1 (s, CH_2), 14.1 (s, CH_2-CH_3).

MS (ES^+) *m/z*: 244.21 ($[MH]^+$, 100%), 214.16 ($[M – C_2H5]^+$, 28), 171.12 ($[M – NEt_2]^+$, 28); Found(ES^+) 244.2054 ($[MH]^+$), $C_{17}H_{26}N^+$ requires 244.2060.

IR (neat) *v*, cm^{-1}: 2920, 2850, 1681, 1447, 1252, 1205, 1072.

(1d)[8]

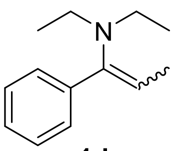

1d

E / *Z* = 91 / 9

A hexane solution (120 mL) of propiophenone (5.0 mL, 37.6 mmol) and diethylamine (24.0 mL, 231 mmol) was cooled to 0 °C and $TiCl_4$ (2.3 mL, 21.0 mmol) was added dropwise and the resulting suspension was left to stir for further 30 minutes followed by stirring the reaction mixture at room temperature for further 24 hours. After this time, the flask was opened to air and water-saturated diethyl ether (23 mL) was added. The solution was filtered and volatiles were removed in vacuo. The product was purified by Kugelrohr distillation (oven T = 85 °C, 1.2 mbar) to afford the desired enamine (2.014 g, 10.6 mmol, 28%) as a pale yellow liquid.

Since the compounds are present as a mixture (*E*/*Z* = 91:9), signals are given to them separately where possible.

^{1}H NMR (400 MHz, C_6D_6, 298 K): δ_H (*E* isomer) = 7.37 – 7.19 (5H, m, Ar-H), 4.53 (1H, q, $^{3}J_{HH}$ = 7.0 Hz, CH), 2.81 (4H, q, $^{3}J_{HH}$ = 7.0 Hz, 2 x N-CH_2), 1.50 (3H, d, $^{3}J_{HH}$ = 6.9 Hz, CH-CH_3), 0.94 (6H, t, $^{3}J_{HH}$ = 7.0 Hz, 2 x CH_3); δ_H (*Z* isomer) = 7.37 – 7.19 (5H, m, Ar-H), 5.08 (1H, q, $^{3}J_{HH}$ = 7.1 Hz, CH), 3.00–2.93 (4H, m, 2 x N-CH_2), 1.73 (3H, d, $^{3}J_{HH}$ = 7.0 Hz, CH-CH_3), 0.99 (6H, t, $^{3}J_{HH}$ = 7.1 Hz, 2 x CH_3).

^{13}C{^{1}H} NMR (101 MHz, $CDCl_3$, 298 K): δ_C (*E* isomer) = 147.4 (s, N-C), 139.0 (s, ^{Ar}C), 129.8 (s, ^{Ar}CH), 127.8 (s, ^{Ar}CH), 126.9 (s, ^{Ar}CH), 99.8 (s, C=CH), 43.0 (s, N-CH_2), 14.0 (s, CH_3), 11.5 (s, N-CH_2-CH_3). ^{13}C NMR signals for *Z*-isomer are not assigned.

MS (ES$^+$) *m/z*: 190.16 ([MH]$^+$, 100%); Found(ES$^+$) 190.1588 ([MH]$^+$), $C_{13}H_{20}N^+$ requires 190.1590.

N,*N*-Diethyl-1-phenylethenamine (**1e**)[9]

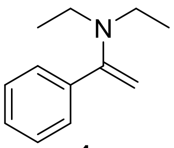

1e

A hexane solution (100 mL) of acetophenone (5.0 mL, 42.9 mmol) and diethylamine (27.0 mL, 260 mmol) was cooled to 0 °C and $TiCl_4$ (2.6 mL, 23.7 mmol) was added dropwise and the resulting suspension was left to stir for further 30 minutes followed by stirring the reaction mixture at room temperature for further 24 hours. After this time, the flask was opened to air and the solution was filtered and volatiles were removed in vacuo. The product was purified by Kugelrohr distillation (oven T = 90 °C, 1.5 mbar) to afford the desired enamine (4.043 g, 23.1 mmol, 54%) as a pale yellow liquid.

^{1}H NMR (300 MHz, C_6D_6, 298 K): δ_H = 7.28 – 7.20 (2H, m, Ar-H), 6.92 – 6.78 (3H, m, Ar-H), 4.13 (1H, s, one of C=CH_2), 3.91 (1H, s, one of C=CH_2), 2.58 (4H, q, $^{3}J_{HH}$ = 7.0 Hz, 2 x N-CH_2), 0.61 (6H, t, $^{3}J_{HH}$ = 7.0 Hz, 2 x CH_3) .

^{13}C{^{1}H} NMR (75 MHz, $CDCl_3$, 298 K): δ_C = 154.4 (s, N-C), 141.6 (s, ^{Ar}C), 128.5 (s, ^{Ar}CH), 128.4 (s, ^{Ar}CH), 128.1 (s, ^{Ar}CH), 90.9 (s, C=CH_2), 43.7 (s, N-CH_2), 12.1 (s, CH_3).

MS (ES$^+$) *m/z*: 176.14 ([MH]$^+$, 100%); Found(ES$^+$) 176.1430 ([MH]$^+$), $C_{12}H_{18}N^+$ requires 176.1434.

(**1f**)

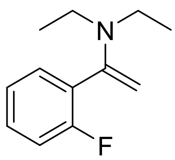

1f

A hexane solution (120 mL) of 1-(2-fluorophenyl)ethan-1-one (5.1 mL, 42.0 mmol) and diethylamine (26.0 mL, 250 mmol) was cooled to 0 °C and $TiCl_4$ (2.53 mL, 23.1 mmol) was added dropwise and the resulting suspension was left to stir for further 30 minutes followed by stirring the reaction mixture at room temperature for further 24 hours. After this time, the flask was opened to air and water-saturated diethyl ether (26 mL) was added. The solution was filtered and volatiles were removed in vacuo. The product was purified by Kugelrohr distillation (oven T = 88 °C, 1.4 mbar) to afford the desired enamine (4.105 g, 21.2 mmol, 51%) as a pale yellow-green liquid.

Surprisingly – this particular enamine is a lot more stable than other enamines with disubstituted double bonds studied here (no hydrolysis detected in wet chloroform after hours).

Anal. Calcd. for $C_{12}H_{16}FN$ (found): C, 74.58 (74.52); H, 8.34 (8.26); N, 7.25 (7.36).

^{1}H NMR (400 MHz, $CDCl_3$, 298 K): δ_H = 7.40 – 7.33 (1H, m, Ar-$\underline{H}$), 7.32 – 7.25 (1H, m, Ar-$\underline{H}$), 7.15 – 7.02 (2H, m, Ar-$\underline{H}$), 4.10 (1H, s, one of C=C$\underline{H_2}$), 3.93 (1H, s, one of C=C$\underline{H_2}$), 3.04 (4H, q, $^3J_{HH}$ = 7.0 Hz, 2 x N-C$\underline{H_2}$), 1.07 (6H, t, $^3J_{HH}$ = 7.0 Hz, 2 x C$\underline{H_3}$) .

^{13}C{^{1}H} NMR (126 MHz, $CDCl_3$, 298 K): δ_C = 160.0 (d, $^1J_{CF}$ = 248 Hz, $^{Ar}\underline{C}$F), 147.9 (s, N-$\underline{C}$), 131.2 (d, $^3J_{CF}$ = 3.7 Hz, $^{Ar}\underline{C}$H), 129.1 (d, $^3J_{CF}$ = 8.1 Hz, $^{Ar}\underline{C}$H), 128.4 (d, $^2J_{CF}$ = 14.7 Hz, $^{Ar}\underline{C}$), 123.7 (d, $^4J_{CF}$ = 3.7 Hz, $^{Ar}\underline{C}$H), 115.5 (d, $^2J_{CF}$ = 22.7 Hz, $^{Ar}\underline{C}$H), 87.9 (s, C=$\underline{C}H_2$), 43.1 (s, N-$\underline{C}H_2$), 12.0 (s, $\underline{C}H_3$).

^{19}F{^{1}H} NMR (377 MHz, $CDCl_3$, 298 K): δ_F = -116.0 (s).

MS (ES^+) *m/z*: 194.13 ($[MH]^+$, 100%); Found(ES^+) 194.1334 ($[MH]^+$), $C_{12}H_{17}FN^+$ requires 194.1340.

IR (neat) *v*, cm^{-1}: 2971, 2864, 1602, 1493, 1440, 1371, 1220.

(1g)

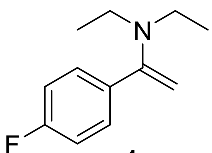

1g

A hexane solution (120 mL) of 1-(4-fluorophenyl)ethan-1-one (5.4 mL, 44.5 mmol) and diethylamine (28.0 mL, 270 mmol) was cooled to 0 °C and $TiCl_4$ (2.7 mL, 24.6 mmol) was added dropwise and the resulting suspension was left to stir for further 30 minutes followed by stirring the reaction mixture at room temperature for further 24 hours. After this time, the flask was opened to air and water-saturated diethyl ether (27 mL) was added. The solution was filtered and volatiles were removed in vacuo. The product was purified by Kugelrohr distillation (oven T = 58 °C, 0.9 mbar) to afford the desired enamine (2.913 g, 15.1 mmol, 34%) as a pale yellow liquid.

^{1}H NMR (300 MHz, C_6D_6, 298 K): δ_H = 7.35 – 7.25 (2H, m, Ar-$\underline{H}$), 6.85 – 6.73 (2H, m, Ar-$\underline{H}$), 4.31 (1H, s, one of C=C$\underline{H_2}$), 4.14 (1H, s, one of C=C$\underline{H_2}$), 2.79 (4H, q, $^{3}J_{HH}$ = 7.0 Hz, 2 x N-C$\underline{H_2}$), 0.87 (6H, t, $^{3}J_{HH}$ = 7.0 Hz, 2 x C$\underline{H_3}$) .

^{13}C{^{1}H} NMR (126 MHz, $CDCl_3$, 298 K): δ_C = 162.6 (d, $^{1}J_{CF}$ = 246 Hz, $^{Ar}\underline{C}$F), 153.6 (s, N-$\underline{C}$), 137.1 (d, $^{4}J_{CF}$ = 3.0 Hz, $^{Ar}\underline{C}$), 129.5 (d, $^{3}J_{CF}$ = 8.0 Hz, $^{Ar}\underline{C}$H), 114.8 (d, $^{2}J_{CF}$ = 21.5 Hz, $^{Ar}\underline{C}$H), 91.3 (s, C=$\underline{C}H_2$), 43.0 (s, N-$\underline{C}H_2$), 11.2 (s, $\underline{C}H_3$).

^{19}F{^{1}H} NMR (470 MHz, $CDCl_3$, 298 K): δ_F = -115.0 (s).

MS (ES$^+$) *m/z*: 194.13 ([MH]$^+$, 100%); Found(ES$^+$) 194.1333 ([MH]$^+$), $C_{12}H_{17}FN^+$ requires 194.1340.

IR (neat) *v*, cm^{-1}: 2973, 1610, 1519, 1384, 1222, 1093.

(1h)

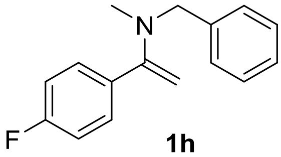

1h

A hexane solution (120 mL) of 1-(4-fluorophenyl)ethan-1-one (5.2 mL, 42.8 mmol) and *N*-methylbenzylamine (33.1 mL, 256 mmol) was cooled to 0 °C and $TiCl_4$ (2.6 mL, 23.7 mmol) was added dropwise and the resulting suspension was left to stir for further 30 minutes followed by stirring the reaction mixture at room temperature for further 24 hours. After this

time, the flask was opened to air and water-saturated diethyl ether (26 mL) was added. The solution was filtered and volatiles were removed in vacuo. The product was purified by distilling impurities of using Kugelrohr (oven T = 95 °C, 1.4 mbar) to afford the desired enamine (5.928 g, 24.3 mmol, 57%) as very viscous brown oil.

^{1}H NMR (300 MHz, C_6D_6, 298 K): δ_H = 7.44 – 7.36 (2H, m, Ar-H), 7.23 – 7.08 (5H, m, Ar-H), 6.84 – 6.74 (2H, m, Ar-H), 4.32 (1H, s, one of C=CH_2), 4.16 (1H, s, one of C=CH_2), 3.81 (2H, s, N-CH_2), 2.41 (3H, s, CH_3).

^{13}C{^{1}H} NMR (126 MHz, $CDCl_3$, 298 K): δ_C = 162.5 (d, $^{1}J_{CF}$ = 246 Hz, ^{Ar}CF), 155.6 (s, N-C), 138.5 (s, ^{Ar}C), 136.0 (d, $^{4}J_{CF}$ = 3.0 Hz, ^{Ar}C), 129.5 (d, $^{3}J_{CF}$ = 8.2 Hz, ^{Ar}CH), 128.2 (s, ^{Ar}CH), 127.8 (s, ^{Ar}CH), 126.9 (s, ^{Ar}CH), 115.0 (d, $^{2}J_{CF}$ = 20.9 Hz, ^{Ar}CH), 90.4 (s, C=CH_2), 56.7 (s, N-CH_2), 37.9 (s, CH_3).

^{19}F{^{1}H} NMR (282 MHz, C_6D_6, 298 K): δ_F = -114.4 (s).

MS (ES^+) *m/z*: 242.13 ($[MH]^+$, 100%); Found(ES^+) 242.1336 ($[MH]^+$), $C_{16}H_{17}FN^+$ requires 242.1340.

IR (neat) *v*, cm^{-1}: 3028, 2799, 1601, 1506, 1366, 1219, 1153.

N-Benzyl-*N*-methyl-1-phenylethenamine (**1i**)[9]

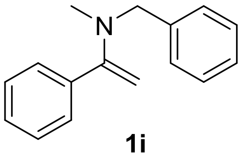

1i

A hexane solution (300 mL) of acetophenone (12.0 mL, 102 mmol) and *N*-methylbenzylamine (79.0 mL, 612 mmol) was cooled to 0 °C and $TiCl_4$ (6.2 mL, 56.5 mmol) was added dropwise and the resulting suspension was left to stir for further 30 minutes followed by stirring the reaction mixture at room temperature for further 24 hours. After this time, the flask was opened to air and water-saturated diethyl ether (62 mL) was added. The solution was filtered and volatiles were removed in vacuo. The product was purified by distilling impurities of using Kugelrohr (oven T = 95 °C, 1.5 mbar) to afford the desired enamine (13.462 g, 60.3 mmol, 59%) as very viscous brown oil.

^{1}H NMR (300 MHz, C_6D_6, 298 K): δ_H = 7.35 – 7.29 (2H, m, Ar-H), 6.92 – 6.74 (8H, m, Ar-H), 4.12 (1H, s, one of C=CH_2), 3.89 (1H, s, one of C=CH_2), 3.56 (2H, s, N-CH_2), 2.13 (3H, s, CH_3).

^{13}C NMR (75 MHz, $CDCl_3$, 298 K): δ_C = 156.9 (s, N-C), 140.3 (s, ^{Ar}C), 138.9 (s, ^{Ar}C), 128.5 – 128.0 (m, ^{Ar}CH), 90.4 (s, C=CH_2), 57.0 (s, N-CH_2), 38.1 (s, CH_3).

MS (ES$^+$) *m/z*: 224.14 ($[MH]^+$, 100%), 122.10 ($[MH_2 – C_8H_7]^+$, 70), 91.05 ($[C_7H_7]^+$, 41).

General hydroaminovinylation of *N*-benzylpent-4-en-1-amine.

A high pressure autoclave with 4 vials was used. A vial was sealed, purged with argon for 10 minutes and charged with a toluene solution of $[Rh(acac)(CO)_2]$ (0.5 mL, 8 mM, containing 4.0 μmol of $[Rh(acac)(CO)_2]$) followed by toluene solution of BOBPHOS ligand (0.5 mL, 10 mM, containing 5.0 μmol of the ligand). This was left to stir for 10 minutes and toluene solution of *N*-benzylpent-4-en-1-amine (1.0 mL, 1 M, contains 1.0 mmol of *N*-benzylpent-4-en-1-amine and 0.1 mL of 1-methylnaphthalene as an internal standard) was added. This was purged with syngas (1:1 mixture) 3 times, pressurised with syngas (10 bar), heated to the desired temperature and left to stir for 16 hours. After this time, the vessel was cooled to room temperature, the pressure was released and a ^{1}H NMR of the crude solution was acquired in order to calculate the conversion.

The reaction was tested at 60 °C (selectivity towards **1j** = 69%), 50 °C (selectivity towards **1j** = 78%) and at 25 °C (selectivity towards **1j** = 68%). Activation of catalyst before the reaction at 50 °C by literature procedure[4] did not make any difference towards selectivity / activity of the catalyst. Single enantiomer of the ligand was tested as well at 50 °C where the same selectivity as with mixture of diastereomers was achieved.

Synthesis of 1-benzyl-5-methyl-1,2,3,4-tetrahydropyridine (**1j**).

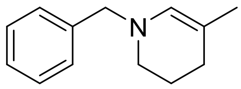

1j

A flask was purged 3 times with vacuum / argon cycles and charged with a toluene solution of $[Rh(acac)(CO)_2]$ (4.0 mL, 8 mM, containing 32.0 μmol of $[Rh(acac)(CO)_2]$) followed by toluene solution of BOBPHOS ligand (4.0 mL, 10 mM, containing 40.0 μmol of the ligand). This was left to stir for 10 minutes and toluene solution of *N*-benzylpent-4-en-1-amine (8.0 mL, 1 M, contains 8.0 mmol of *N*-benzylpent-4-en-1-amine) was added. The flask was charged to the high pressure autoclave, the autoclave was purged with syngas (1:1 mixture) 3 times, pressurised with syngas (10 bar), heated to 50 °C and left to stir for 16 hours. After this time, the vessel was cooled to room temperature, the pressure was released and a ^{1}H NMR of

the crude solution was acquired in order to calculate the conversion. The product was isolated by Kugelrohr distillation (oven T = 110 °C, 1.5 mbar) as a pale yellow liquid (0.900 g, 4.81 mmol, 60%).

^{1}H NMR (300 MHz, $CDCl_3$, 298 K): δ_H = 7.32-7.10 (5H, m, Ar-H), 5.67 (1H, br s, N-CH), 3.77 (2H, s, Ar-CH_2), 2.70-2.62 (2H, m, N-CH_2), 1.91-1.71 (4H, m, 2 x CH_2), 1.52 (3H, s, CH_3).

^{13}C NMR (75 MHz, $CDCl_3$, 298 K): δ_C = 139.2 (s, ^{Ar}C), 131.8 (s, N-CH), 128.8 (s, ^{Ar}CH), 128.7 (s, ^{Ar}CH), 127.4 (s, ^{Ar}CH), 107.4 (s, CH_3-C), 60.4 (s, ^{Ar}C-CH_2), 47.8 (s, N-CH_2), 27.1 (s, CH_2), 23.1 (s, CH_2), 21.4 (s, CH_3).

MS (ES^+) *m/z*: 188.14 ($[MH]^+$, 100%), 176.14 ($[M – CH]^+$, 26), 91.05 ($[C_7H_7]^+$, 23).

Hydrogenation of enamines.

General procedure for enamine hydrogenation.

A high pressure autoclave with 4 vials was used. A vial was charged with the desired ligand (8.0 µmol), sealed and purged with Ar for 10 minutes. The ligand was dissolved in toluene (0.5 mL) and a toluene solution of $[Rh(COD)Cl]_2$ (0.5 mL, 4 mM, containing 2.0 µmol of $[Rh(COD)Cl]_2$) was added. This was left to stir for 10 minutes before a toluene solution of the desired enamine (1.0 mL, 1 M, contains 1.0 mmol of enamine and 0.1 mL of 1-methylnaphthalene) was added and the resulting solution was stirred for 5 minutes. After this time, the vial placed into the pre-purged autoclave, the autoclave was sealed, purged with hydrogen 3 times, pressurised with H_2 gas to the desired pressure, heated to 65 °C and left to stir for 16 hours. After this time, the autoclave was cooled to room temperature, the gas pressure was released and ^{1}H NMR of the crude reaction solution was acquired in order to calculate the conversion.

Isolation of amines.

General procedure (**2b, 2d–2j**).

After catalytic hydrogenation of enamine (only in cases of full conversions to the amines), the solution was diluted with toluene (8 mL) and the amine was extracted with hydrochloric acid (1 M, 3 × 20 mL). Combined acid fractions were basified with aq. NaOH (1 M) until pH 12, and the amine was extracted with ethyl acetate (3 × 25 mL). Combined organic fractions

were washed with brine (30 mL), dried over $MgSO_4$ and solvent was removed under reduced pressure. The desired product was dried in vacuo for 50 minutes to afford the desired amine.

(**2b**)

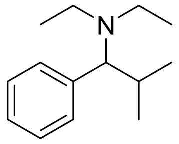

2b

The product is a pale-yellow oil (152 mg, 74.0 mmol, 74%).

^{1}H NMR (300 MHz, $CDCl_3$, 298 K): δ_H = 7.34 – 7.19 (3H, m, Ar-H), 7.17 – 7.11 (2H, m, Ar-H), 3.22 (1H, d, $^3J_{HH}$ = 9.5 Hz, N-CH), 2.70 – 2.55 (2H, m, CH_2), 2.30 – 2.16 (1H, m, CH), 2.15 – 2.01 (2H, m, CH_2), 1.06 – 0.98 (9H, m, 3 x CH_3), 0.70 (3H, d, $^3J_{HH}$ = 6.5 Hz, CH-CH_3).

^{13}C NMR (75 MHz, $CDCl_3$, 298 K): δ_C = 138.9 (^{Ar}C), 129.2 (s, ^{Ar}CH), 127.5 (s, ^{Ar}CH), 126.4 (s, ^{Ar}CH), 70.6 (s, N-CH), 42.7 (s, CH_2), 28.6 (s, CH), 20.9 (s, CH-CH_3), 20.1 (s, CH-CH_3), 13.3 (s, CH_2-CH_3).

MS (ES^+) *m/z*: 206.19 ($[MH]^+$, 100%), 133.10 ($[M - NEt_2]^+$, 21); Found(ES^+) 206.1901 ($[MH]^+$), $C_{14}H_{24}N^+$ requires 206.1903.

IR (neat) *v*, cm^{-1}: 2966, 2812, 1450, 1381, 1051.

(**2c**)

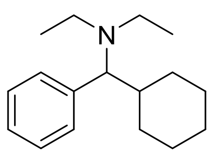

2c

Since conversion was only 67%, after acid-basic work-up the amine was obtained as a 2:1 mixture with enamine (due to high stability of enamine, it was not hydrolysed). The mixture was dissolved in ethyl acetate and passed through a short column of silica (eluting solvent is EtOAc:Petroleum ether (40–60):Et_3N as 1:3:0.08 mixture). This allowed all enamine to form some new compounds. After this, the solvents were removed under reduced pressure and the

mixture was purified by a silica column (eluting solvent is EtOAc:Petroleum ether (40–60):Et_3N as 1:14:0.08 mixture; R_f = 0.13) to afford the desired amine (77 mg, 31.4 mmol, 47%) as a colourless viscous oil.

^{1}H NMR (400 MHz, $CDCl_3$, 298 K): δ_H = 7.36 – 7.22 (3H, m, Ar-H), 7.19 – 7.13 (2H, m, Ar-H), 3.37 (1H, d, $^3J_{HH}$ = 9.7 Hz, N-CH), 2.71 – 2.60 (2H, m, N-CH_2), 2.20 – 2.05 (3H, m,CH and N-CH_2), 1.98 – 1.87 (1H, m, one of CH_2), 1.85 – 1.76 (1H, m, one of CH_2), 1.72 – 1.60 (2H, m, CH_2), 1.47 – 1.12 (4H, m, 2 x CH_2), 1.05 (6H, t, $^3J_{HH}$ = 7.1 Hz, 2 x CH_3), 1.00 – 0.87 (1H, m, one of CH_2), 0.82 – 0.70 (1H, m, one of CH_2).

^{13}C NMR (101 MHz, $CDCl_3$, 298 K): δ_C = 138.7 (^{Ar}C), 129.2 (s, ^{Ar}CH), 127.5 (s, ^{Ar}CH), 126.4 (s, ^{Ar}CH), 69.3 (s, N-CH), 42.8 (s, N-CH_2), 38.3 (s, CH), 31.2 (s, CH_2), 30.8 (s, CH_2), 26.9 (s, CH_2), 26.4 (s, CH_2), 26.3 (s, CH_2), 13.4 (s, CH_2-CH_3).

MS (ES^+) *m/z*: 246.22 ($[MH]^+$, 100%), 173.13 ($[M - NEt_2]^+$, 84); Found(ES^+) 246.2207 ($[MH]^+$), $C_{17}H_{28}N^+$ requires 246.2216.

IR (neat) *v*, cm^{-1}: 2972, 2783, 1492, 1448, 1159.

(2d)

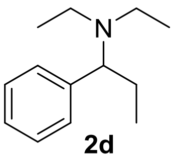

2d

The product is a pale-yellow oil (174 mg, 90.9 mmol, 91%).

^{1}H NMR (300 MHz, $CDCl_3$, 298 K): δ_H = 7.34 – 7.19 (5H, m, Ar-H), 3.51 (1H, dd, $^3J_{HH}$ = 9.5 Hz, $^3J_{HH}$ = 5.1 Hz, N-CH), 2.72 – 2.58 (2H, m, N-CH_2), 2.44 – 2.29 (2H, m, N-CH_2), 2.00 – 1.84 (1H, m, one from CH_2), 1.80 – 1.63 (1H, m, one from CH_2), 1.00 (6H, t, $^3J_{HH}$ = 7.1 Hz, 2 x N-CH_2-CH_3), 0.76 (3H, t, $^3J_{HH}$ = 7.3 Hz, CH_3).

^{13}C NMR (101 MHz, $CDCl_3$, 298 K): δ_C = 141.8 (^{Ar}C), 128.7 (s, ^{Ar}CH), 127.9 (s, ^{Ar}CH), 126.6 (s, ^{Ar}CH), 66.5 (s, N-CH), 43.1 (s, N-CH_2), 25.8 (s, CH), 12.5 (s, N-CH_2-CH_3), 11.4 (s, CH_3).

MS (ES^+) *m/z*: 192.17 ($[MH]^+$, 100%); Found(ES^+) 192.1747 ($[MH]^+$), $C_{13}H_{22}N^+$ requires 192.1747.

IR (neat) *v*, cm^{-1}: 2966, 2810, 1450, 1379, 1196, 1051.

N,*N*-Diethyl-1-phenylethanamine (**2e**)[9]

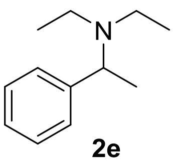

2e

The product is a pale-yellow oil (148 mg, 83.5 mmol, 83%).

^{1}H NMR (300 MHz, $CDCl_3$, 298 K): δ_H = 7.31 – 7.07 (5H, m, Ar-$\underline{H}$), 3.70 (1H, q, $^{3}J_{HH}$ = 6.7 Hz, C$\underline{H}$), 2.57 – 2.33 (4H, m, 2 x C$\underline{H_2}$), 1.25 (3H, d, $^{3}J_{HH}$ = 6.7 Hz, CH-C$\underline{H_3}$), 0.90 (6H, t, $^{3}J_{HH}$ = 7.1 Hz, 2 x -CH_2-C$\underline{H_3}$).

^{13}C NMR (75 MHz, $CDCl_3$, 298 K): δ_C = 145.3 ($^{Ar}\underline{C}$), 128.1 (s, $^{Ar}\underline{C}$H), 127.6 (s, $^{Ar}\underline{C}$H), 126.5 (s, $^{Ar}\underline{C}$H), 59.2 (s, N-$\underline{C}$H), 42.9 (s, $\underline{CH_2}$), 18.5 (s, CH-$\underline{CH_3}$), 12.2 (s, CH_2-$\underline{CH_3}$).

MS (ES^{+}) *m/z*: 178.16 ([MH]$^{+}$, 86%), 105.07 ([M – NEt_2]$^{+}$, 100); Found(ES^{+}) 178.1586 ([MH]$^{+}$), $C_{12}H_{20}N^{+}$ requires 178.1590.

(**2f**)

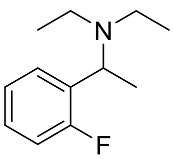

2f

The product is a pale-yellow oil (107 mg, 54.8 mmol, 55%).

^{1}H NMR (300 MHz, $CDCl_3$, 298 K): δ_H = 7.48 – 7.40 (1H, m, Ar-$\underline{H}$), 7.23 – 7.14 (1H, m, Ar-$\underline{H}$), 7.13 – 7.06 (1H, m, Ar-$\underline{H}$), 7.04 – 6.95 (1H, m, Ar-$\underline{H}$), 4.19 (1H, q, $^{3}J_{HH}$ = 6.8 Hz, C$\underline{H}$), 2.70 – 2.43 (4H, m, 2 x C$\underline{H_2}$), 1.35 (3H, d, $^{3}J_{HH}$ = 6.8 Hz, CH-C$\underline{H_3}$), 1.00 (6H, t, $^{3}J_{HH}$ = 7.1 Hz, 2 x -CH_2-C$\underline{H_3}$).

^{19}F{^{1}H} NMR (282 MHz, $CDCl_3$, 298 K): δ_F = -119.1 (s).

^{13}C{^{1}H} NMR (75 MHz, $CDCl_3$, 298 K): δ_C = 160.8 (d, $^{1}J_{CF}$ = 245 Hz, $^{Ar}\underline{C}$F), 131.6 (d, $^{2}J_{CF}$ = 13.6 Hz, $^{Ar}\underline{C}$), 128.7 (d, $^{3}J_{CF}$ = 4.9 Hz, $^{Ar}\underline{C}$H), 127.8 (d, $^{3}J_{CF}$ = 8.4 Hz, $^{Ar}\underline{C}$H), 123.8 (d, $^{4}J_{CF}$ = 3.5 Hz, $^{Ar}\underline{C}$H), 115.1 (d, $^{2}J_{CF}$ = 23.3 Hz, $^{Ar}\underline{C}$H), 51.8 (s, N-$\underline{C}$), 42.8 (s, N-$\underline{C}H_2$), 19.2 (s, CH-$\underline{C}H_3$), 11.8 (s, CH_2-$\underline{C}H_3$).

MS (ES^{+}) *m/z*: 196.15 ([MH]$^{+}$, 100%), 123.06 ([M – NEt_2]$^{+}$, 40); Found(ES^{+}) 196.1492 ([MH]$^{+}$), $C_{12}H_{20}N^{+}$ requires 196.1496.

IR (neat) *v*, cm^{-1}: 2951, 2789, 1452, 1360, 1094, 1011.

(**2g**)

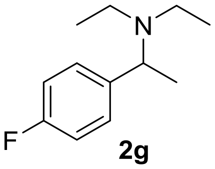

2g

The product is a pale-yellow oil (111 mg, 56.8 mmol, 57%).

^{1}H NMR (300 MHz, $CDCl_3$, 298 K): δ_H = 7.36 – 7.27 (2H, m, Ar-H), 7.03 – 6.92 (2H, m, Ar-H), 3.77 (1H, q, $^3J_{HH}$ = 6.8 Hz, CH), 2.62 – 2.40 (4H, m, 2 x CH_2), 1.30 (3H, d, $^3J_{HH}$ = 6.7 Hz, CH-CH_3), 0.98 (6H, t, $^3J_{HH}$ = 7.1 Hz, 2 x -CH_2-CH_3).

^{19}F{^{1}H} NMR (282 MHz, $CDCl_3$, 298 K): δ_F = -117.4 (s).

^{13}C{^{1}H} NMR (75 MHz, $CDCl_3$, 298 K): δ_C = 161.6 (d, $^1J_{CF}$ = 244 Hz, ^{Ar}CF), 141.0 (d, $^4J_{CF}$ = 3.1 Hz, ^{Ar}C), 128.9 (d, $^3J_{CF}$ = 7.7 Hz, ^{Ar}CH), 114.7 (d, $^2J_{CF}$ = 21.0 Hz, ^{Ar}CH), 58.4 (s, N-C), 42.7 (s, N-CH_2), 18.2 (s, CH-CH_3), 12.1 (s, CH_2-CH_3).

MS (ES^+) *m/z*: 196.15 ($[MH]^+$, 100%), 123.06 ($[M – NEt_2]^+$, 16); Found(ES^+) 196.1492 ($[MH]^+$), $C_{12}H_{20}N^+$ requires 196.1496.

IR (neat) *v*, cm^{-1}: 2968, 2810, 1602, 1506, 1221, 1074.

(**2h**)

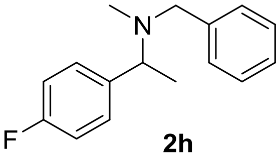

2h

The product is a very viscous yellow oil (214 mg, 87.9 mmol, 88%).

^{1}H NMR (300 MHz, $CDCl_3$, 298 K): δ_H = 7.34 – 7.10 (7H, m, Ar-H), 7.00 – 6.89 (2H, m, Ar-H), 3.55 (1H, q, $^3J_{HH}$ = 6.7 Hz, N-CH), 3.47 (1H, d, $^2J_{HH}$ = 13.3 Hz, one of CH_2), 3.23 (1H, d, $^2J_{HH}$ = 13.3 Hz, one of CH_2), 2.04 (3H, s, N-CH_3), 1.32 (3H, d, $^3J_{HH}$ = 6.7 Hz, CH-CH_3).

^{13}C NMR (75 MHz, $CDCl_3$, 298 K): δ_C = 161.7 (d, $^1J_{CF}$ = 244 Hz, ^{Ar}CF), 140.0-139.8 (m, two quarternary ^{Ar}C), 129.0 (d, $^3J_{CF}$ = 7.8 Hz, ^{Ar}CH), 128.7 (s, ^{Ar}CH), 128.2 (s, ^{Ar}CH), 126.8

(s, $^{Ar}\underline{C}H$), 114.9 (d, $^2J_{CF}$ = 21.0 Hz, $^{Ar}\underline{C}H$), 62.3 (s, N-$\underline{C}$), 58.7 (s, $\underline{C}H_2$), 38.2 (s, N-$\underline{C}H_3$), 18.1 (s, CH-$\underline{C}H_3$).

$^{19}F\{^1H\}$ NMR (282 MHz, $CDCl_3$, 298 K): δ_F = -116.9 (s).

MS (ES^+) *m/z*: 244.15 ($[MH]^+$, 100%); Found(ES^+) 244.1488 ($[MH]^+$), $C_{16}H_{19}FN^+$ requires 244.1496.

IR (neat) *v*, cm^{-1}: 2972, 2785, 1601, 1506, 1494, 1452, 1219, 1153.

N-Benzyl-*N*-methyl-1-phenylethanamine (**2i**)[9]

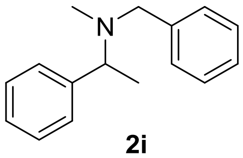

2i

The product is a very viscous yellow oil (206 mg, 91.4 mmol, 91%).

^1H NMR (300 MHz, $CDCl_3$, 298 K): δ_H = 7.36 – 7.09 (10H, m, Ar-$\underline{H}$), 3.55 (1H, q, $^3J_{HH}$ = 6.7 Hz, N-C$\underline{H}$), 3.49 (1H, d, $^2J_{HH}$ = 13.2 Hz, one of C$\underline{H_2}$), 3.21 (1H, d, $^2J_{HH}$ = 13.2 Hz, one of C$\underline{H_2}$), 2.05 (3H, s, N-C$\underline{H_3}$), 1.33 (3H, d, $^3J_{HH}$ = 6.7 Hz, CH-C$\underline{H_3}$).

^{13}C NMR (75 MHz, $CDCl_3$, 298 K): δ_C = 144.1 (s, $^{Ar}\underline{C}$), 140.0 (s, $^{Ar}\underline{C}$), 128.7 (s, $^{Ar}\underline{C}H$), 128.2 (2 x C, s, $^{Ar}\underline{C}H$), 127.7 (s, $^{Ar}\underline{C}H$), 126.8 (s, $^{Ar}\underline{C}H$), 126.7 (s, $^{Ar}\underline{C}H$), 63.2 (s, N-$\underline{C}H$) , 58.8 (s, $\underline{C}H_2$) , 38.3 (s, N-$\underline{C}H_3$) , 18.4 (s, CH-$\underline{C}H_3$).

MS (ES^+) *m/z*: 226.16 ($[MH]^+$, 100%).

1-Benzyl-3-methylpiperidine (**2j**)

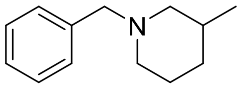

2j

The product is a yellow oil (0.140 g, 74.0 mmol, 74% yield).

^1H NMR (300 MHz, $CDCl_3$, 298 K): δ_H = 7.27 – 7.02 (5H, m, Ar-$\underline{H}$), 3.38 (2H, s, Ar-C$\underline{H_2}$), 2.76 – 2.64 (2H, m, C$\underline{H_2}$), 1.82 – 1.70 (1H, m, Alkyl-$\underline{H}$), 1.65 – 1.41 (5H, m, Alkyl-$\underline{H}$s), 0.84 – 0.67 (1H, m, Alkyl-$\underline{H}$), 0.74 (3H, d, $^3J_{HH}$ = 6.2 Hz, C$\underline{H_3}$).

$^{13}C\{^1H\}$ NMR (75 MHz, $CDCl_3$, 298 K): δ_C = 138.2 (s, $^{Ar}\underline{C}$), 129.1 (s, $^{Ar}\underline{C}H$), 128.0 (s, $^{Ar}\underline{C}H$), 126.8 (s, $^{Ar}\underline{C}H$), 63.4 (s, ^{Ar}C-$\underline{C}H_2$), 61.7 (s, $\underline{C}H_2$), 53.8 (s, $\underline{C}H_2$), 32.9 (s, CH-$\underline{C}H_2$), 30.9 (s, $\underline{C}H$), 25.3 (s, $\underline{C}H_2$), 19.6 (s, $\underline{C}H_3$).

MS (ES^+) *m/z*: 190.16 ($[MH]^+$, 100%); Found(ES^+) 190.1587 ($[MH]^+$), $C_{13}H_{20}N^+$ requires 190.1590.

Reactions in (*R*)-limonene as a solvent.

A high pressure autoclave with 4 vials was used. A vial was charged with $[Rh(COD)Cl]_2$ (1.0 mg, 2.0 μmol) and the desired ligand (8.0 μmol), sealed and purged with Ar for 10 minutes. A (*R*)-limonene solution of the desired enamine (2.0 mL, 0.5 M, contains 1.0 mmol of enamine, 6.7 mmol of solvent and 0.1 mL of 1-methylnaphthalene) was added and the resulting solution was stirred for 5 minutes. After this time, the vial placed into the pre-purged autoclave, the autoclave was sealed, purged with hydrogen 3 times, pressurised with H_2 gas to the desired pressure, heated to the desired temperature and left to stir for 16 hours. After this time, the autoclave was cooled to room temperature, the gas pressure was released and ^1H NMR of the crude reaction solution was acquired in order to calculate the conversion (see Table 1 below – for scheme see Table 3 in the paper).

Table 1. Hydrogenation of enamines in (*R*)-limonene

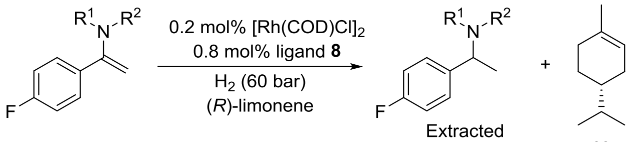

Entry[a]	Enamine	Ligand	Time, h	T, °C	Amine, %[b]	**10**, %[b]
1[c]	**1g**	**4**	16	65	34	> 99
2[c]	**1g**	**8**	16	65	> 99	91
3	**1h**	**4**	16	40	0	48
4	**1h**	**8**	16	40	74	20
5	**1h**	**4**	20	45	11	65
6	**1h**	**8**	20	45	92	23

[a]General conditions: 1.5 mmol of **1h**, 0.2 mol % of $[Rh(COD)Cl]_2$, 0.8 mol % ligand, 0.15 mL of 1-methylnaphthalene as an internal standard, 60 bar of H_2 gas, (*R*)-limonene. Ratio of solvent/enamine = 6.68:1. [b]Determined by ^1H NMR relative to 1-methylnaphthalene. [c]Ratio of solvent/enamine = 6.74:1.

Amine products from entries 2 and 6 were isolated by the following procedure:

The solution was diluted with (*R*)-limonene (8 mL) and the amine was extracted with hydrochloric acid (1 M, 3 × 20 mL). The combined aqueous fractions were basified with aq. NaOH (1 M) until pH 12, and the amine was extracted with ethyl acetate (3 × 25 mL). The

combined organic fractions were washed with brine (30 mL), dried over $MgSO_4$ and the solvent was removed under reduced pressure. The desired product was dried in vacuo for 50 minutes to afford the desired amine. Purity of **2g** is >99%; and the product from entry 6 was isolated as a 9:1 mixture of **2h** and *N*-methylbenzylamine respectively due to the ~90% conversion of the enamine. In order to confirm that the chiral solvent doesn't result in any asymmetric induction, the ee for entries 2 and 6 was measured by addition of 1.2 equivalents of (*R*)-α-methoxyphenylacetic acid to isolated samples of amines, and ^{1}H NMR spectra were acquired; enantiomeric excess was found to be 0%.

References.

1. T. G. Schenck, J. M. Downes, C. R. C. Milne, P. B. Mackenzie, H. Boucher, J. Whelan, B. Bosnich, *Inorg. Chem.*, 1985, **24**, 2334.
2. R.A. Baber, M.L. Clarke, K. Heslop, A. Marr, A.G. Orpen, P.G. Pringle and A.M. Ward, D.A. Zambrano-Williams, *Dalton Trans.* 2005, 1079.
3. M.L. Clarke, D. Ellis, K.L. Mason, A.G. Orpen, P.G. Pringle, R.L. Wingad, D.A. Zaher and R.T. Baker, *Dalton Trans.* 2005, 1294.
4. G. M. Noonan, J. A. Fuentes, C. J. Cobley, M. L. Clarke, *Angew. Chem. Int. Ed.,* 2012, **51**, 2477.
5. D. V. Gribkov, K. C. Hultzsch, *Angew. Chem. Int. Ed.*, 2004, **43**, 5542.
6. W. A. White, H. Weingarten, *J. Org. Chem.*, 1967, **32**, 213.
7. P. Fang, X.-L. Hou, *Org. Lett.*, 2009, **11**, 4612.
8. X. Zeng, G.D. Frey, S. Kousar, G. Bertrand, *Chem. Eur. J.*, 2009, **15**, 3056.
9. N.E. Lee, S.L. Buchwald, *J. Am. Chem. Soc.*, 1994, **116**, 5985.

NMR spectra.

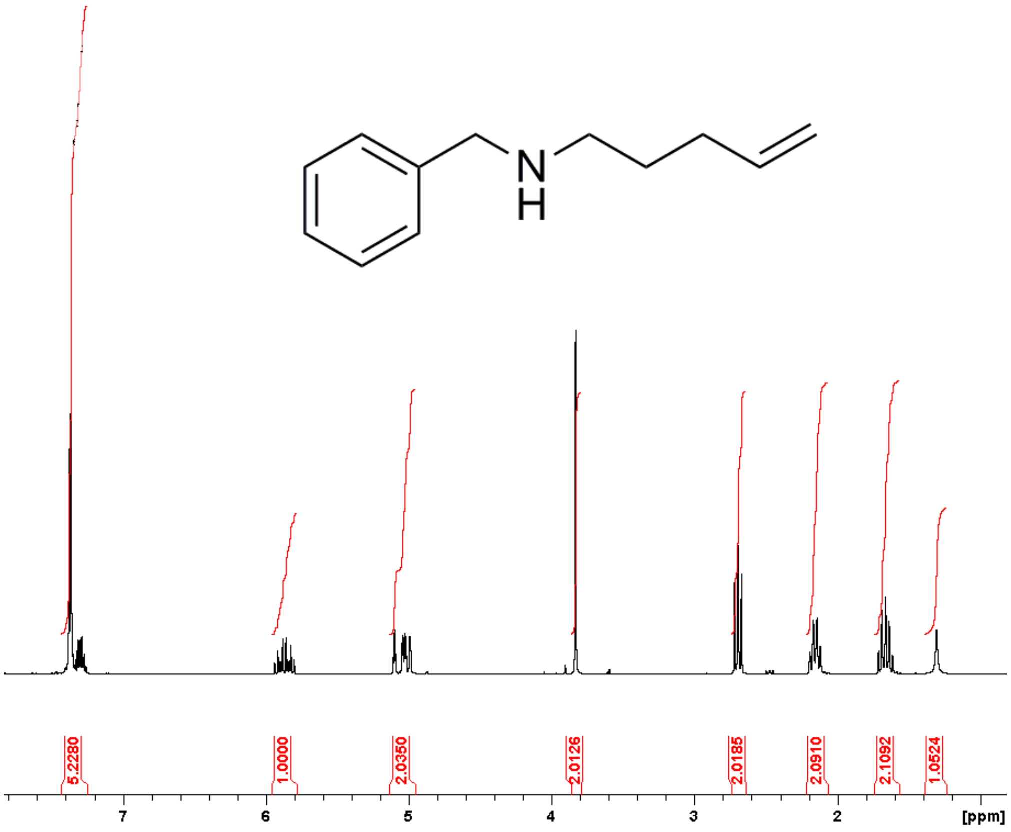

N
H
5.2280
1.0000
2.0350
2.0126
2.0185
2.0910
2.1092
1.0524
7
6
5
4
3
2
[ppm]

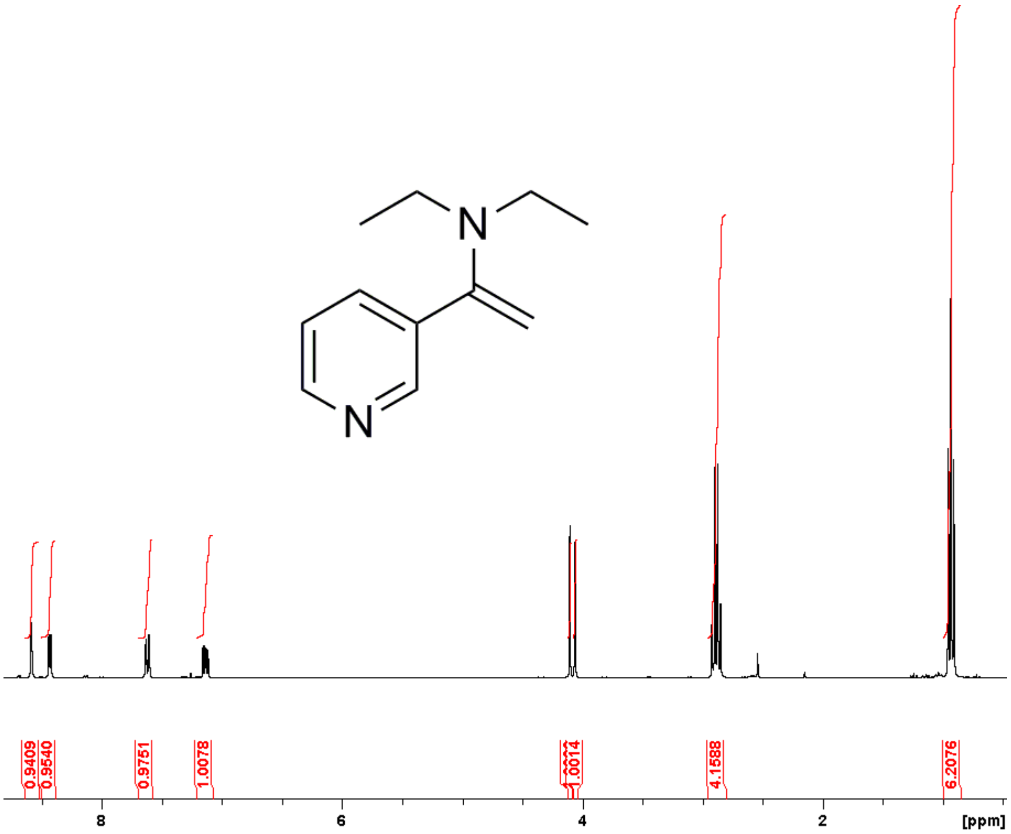

N
N
0.9409
0.9540
0.9751
1.0078
1.0014
4.1588
6.2076
8
6
4
2
[ppm]

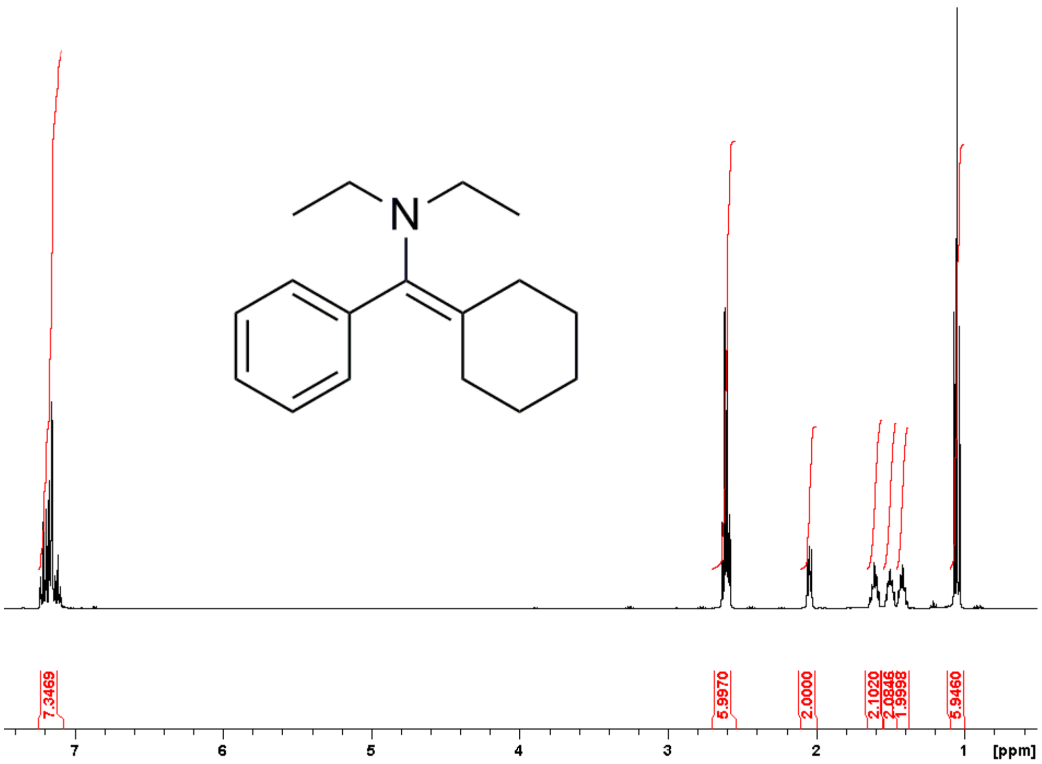
N
7.3469
5.9970
2.0000
2.1020
2.0846
1.9998
5.9460
7
6
5
4
3
2
1
[ppm]

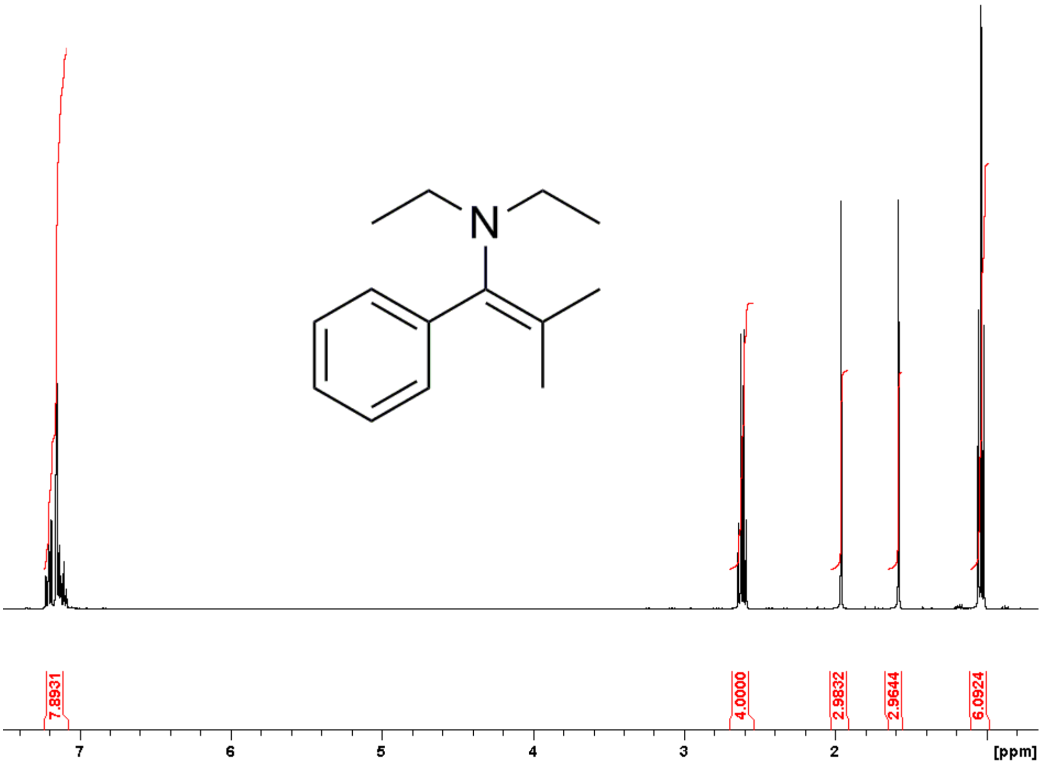
N
7.8931
4.0000
2.9832
2.9644
6.0924
7
6
5
4
3
2
[ppm]

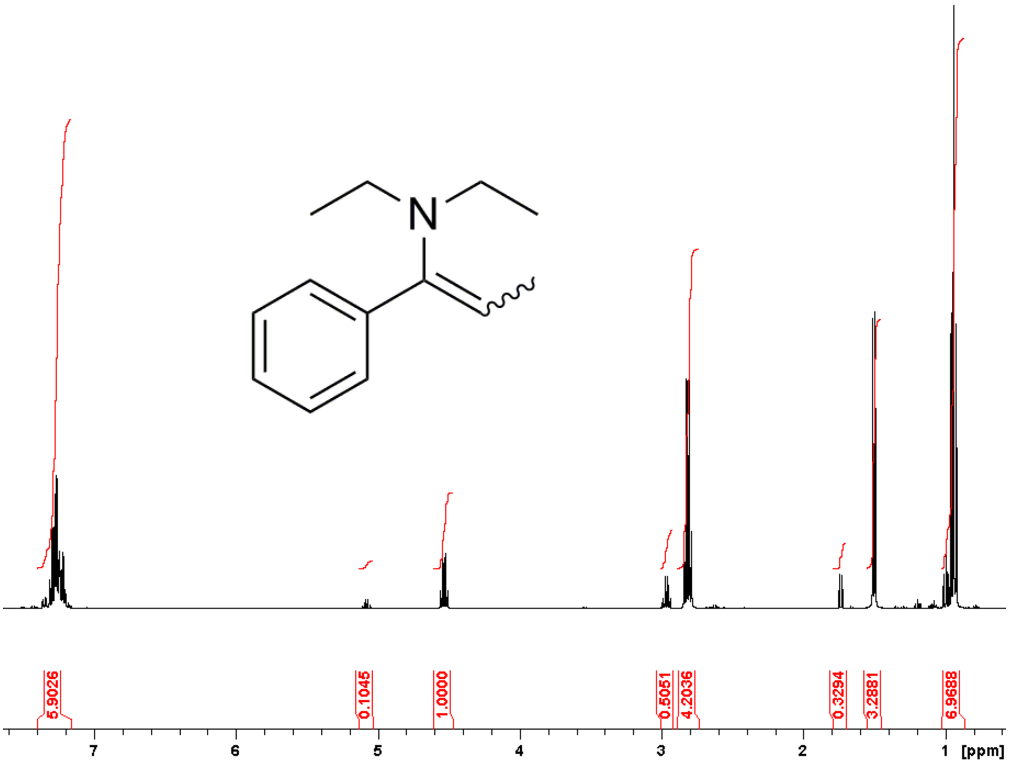
N
5.9026
0.1045
1.0000
0.5051
4.2036
0.3294
3.2881
6.9688
7
6
5
4
3
2
1 [ppm]

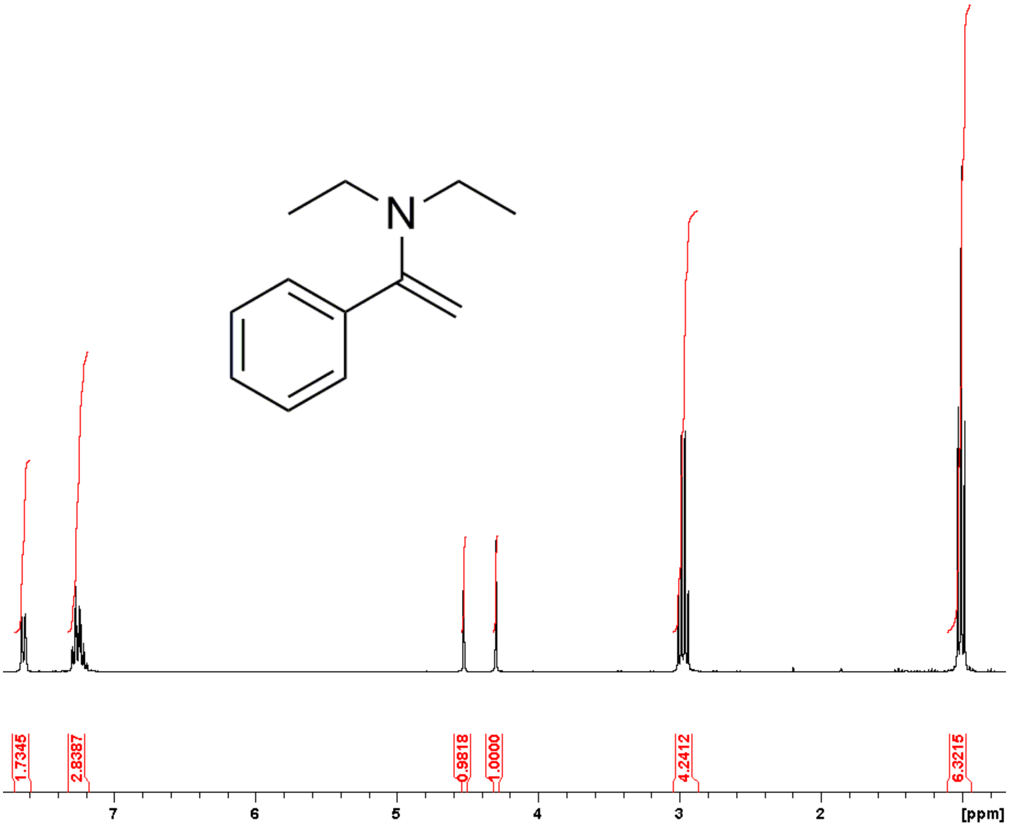
N
1.7345
2.8387
0.9818
1.0000
4.2412
6.3215
7
6
5
4
3
2
[ppm]

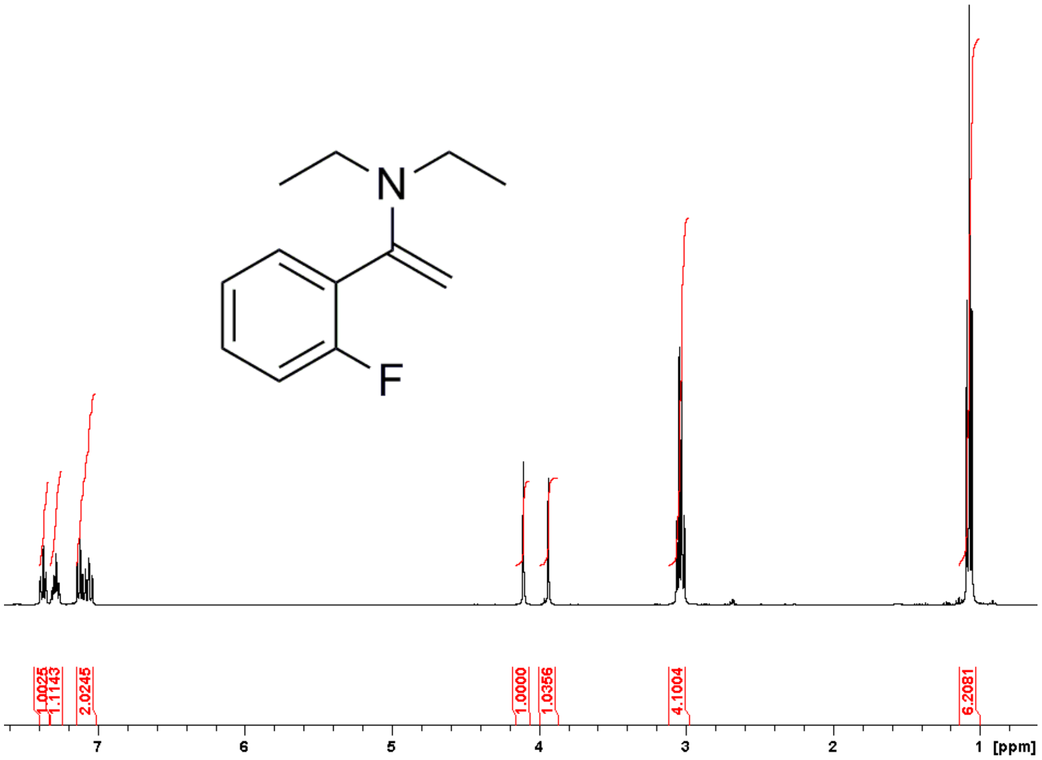
N
F
1.0025
1.1143
2.0245
1.0000
1.0356
4.1004
6.2081
7
6
5
4
3
2
1 [ppm]

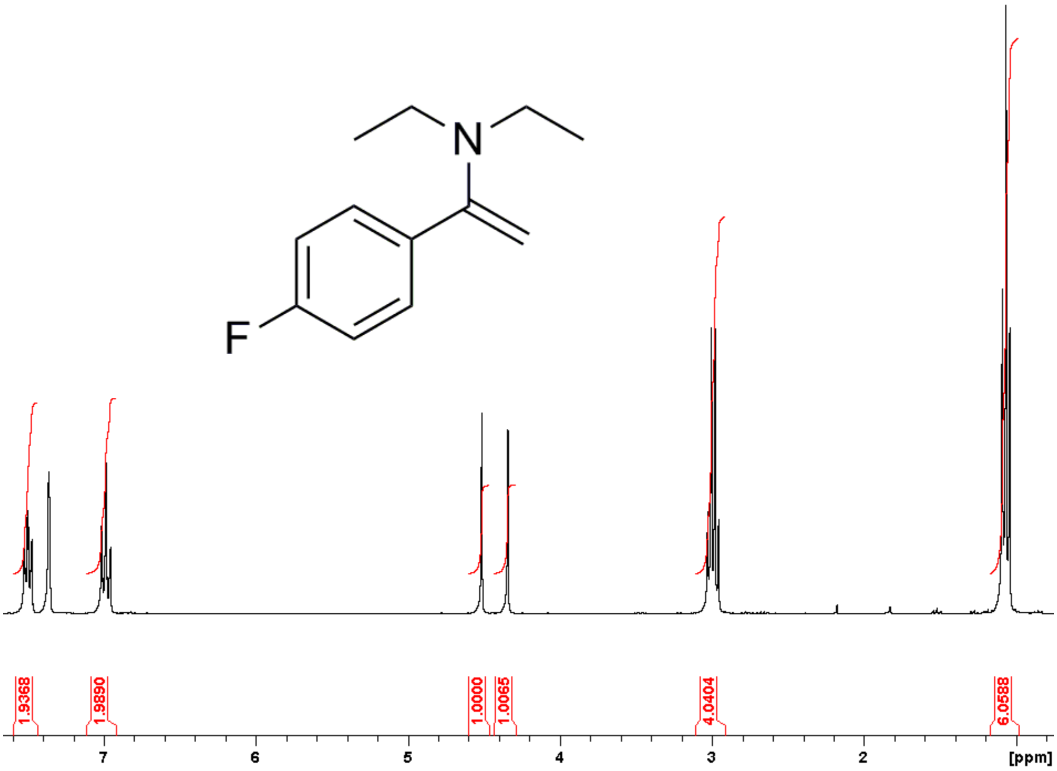
N
F
1.9368
1.9890
1.0000
1.0065
4.0404
6.0588
7
6
5
4
3
2
[ppm]

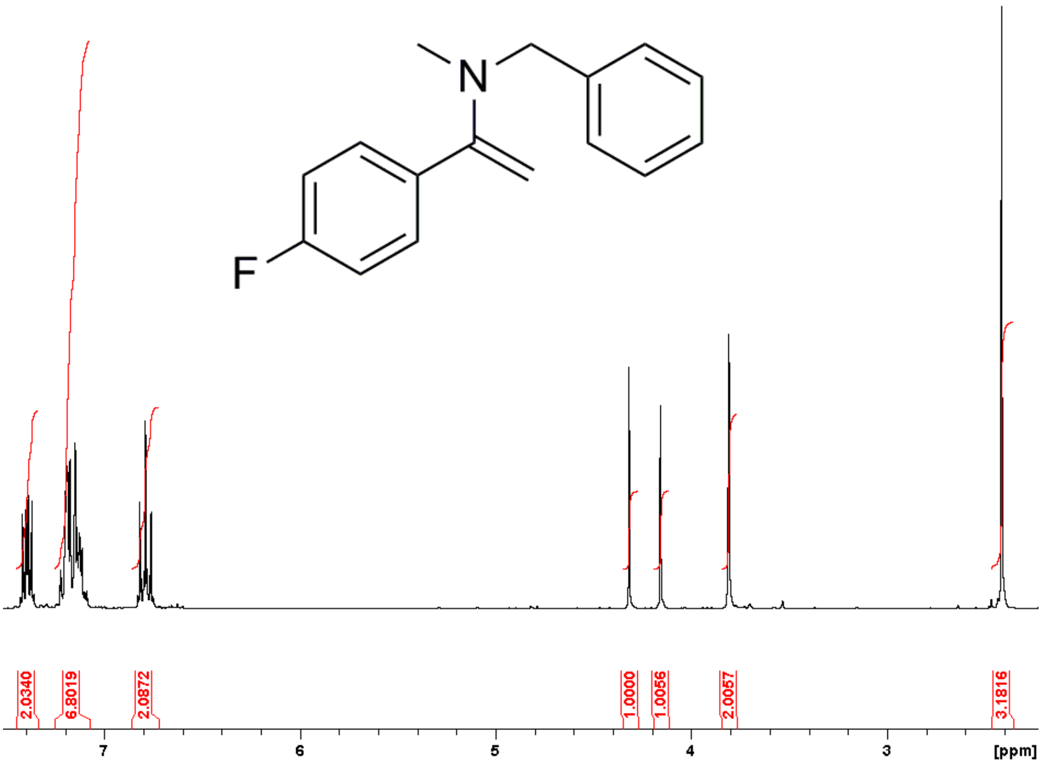
F
N
2.0340
6.8019
2.0872
1.0000
1.0056
2.0057
3.1816
7
6
5
4
3
[ppm]

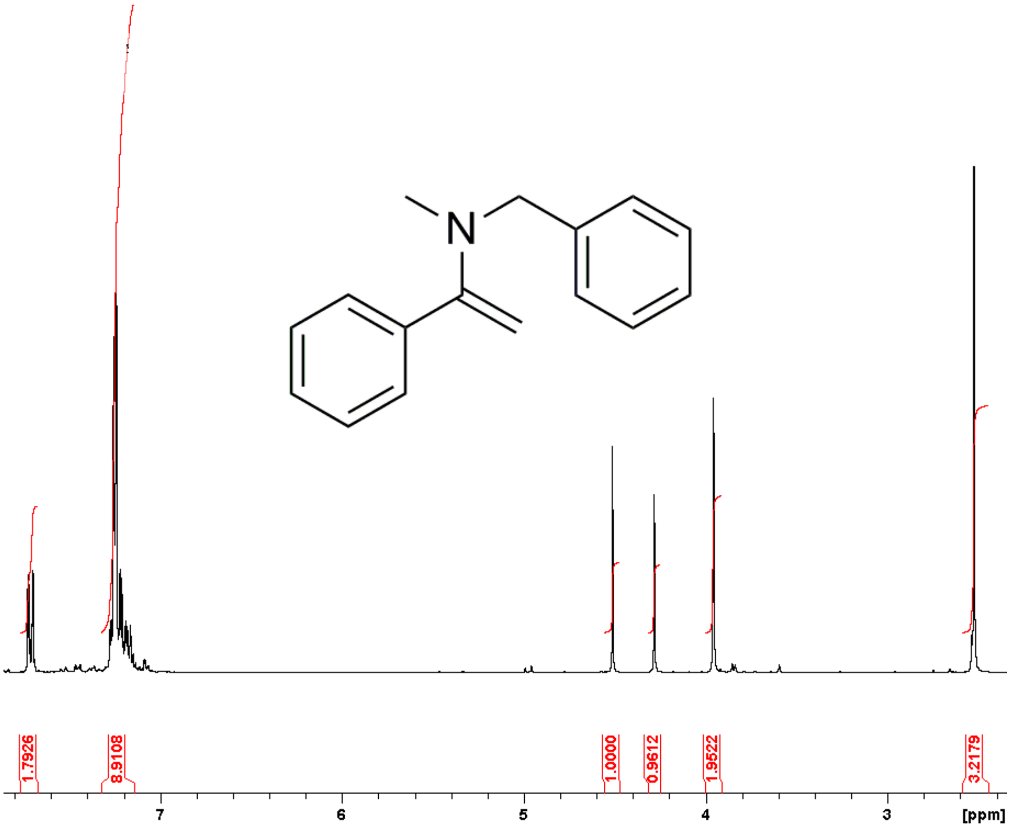
N
1.7926
8.9108
1.0000
0.9612
1.9522
3.2179
7
6
5
4
3
[ppm]

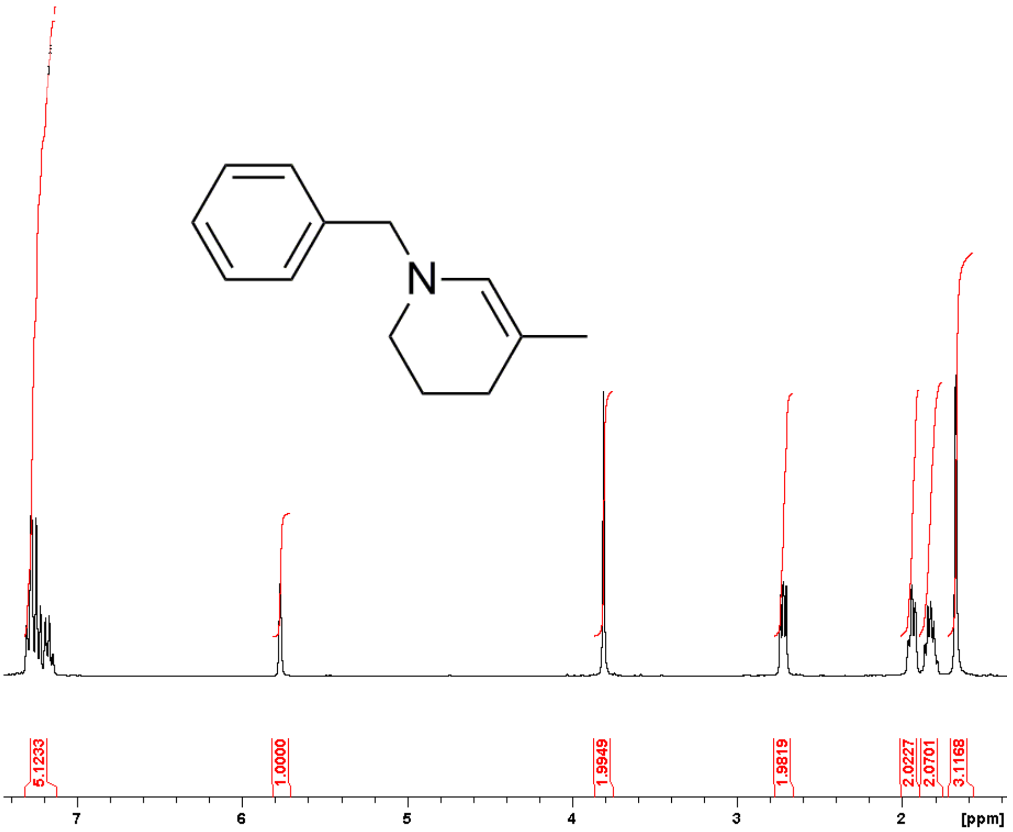
N
5.1233
1.0000
1.9949
1.9819
2.0227
2.0701
3.1168
7
6
5
4
3
2
[ppm]

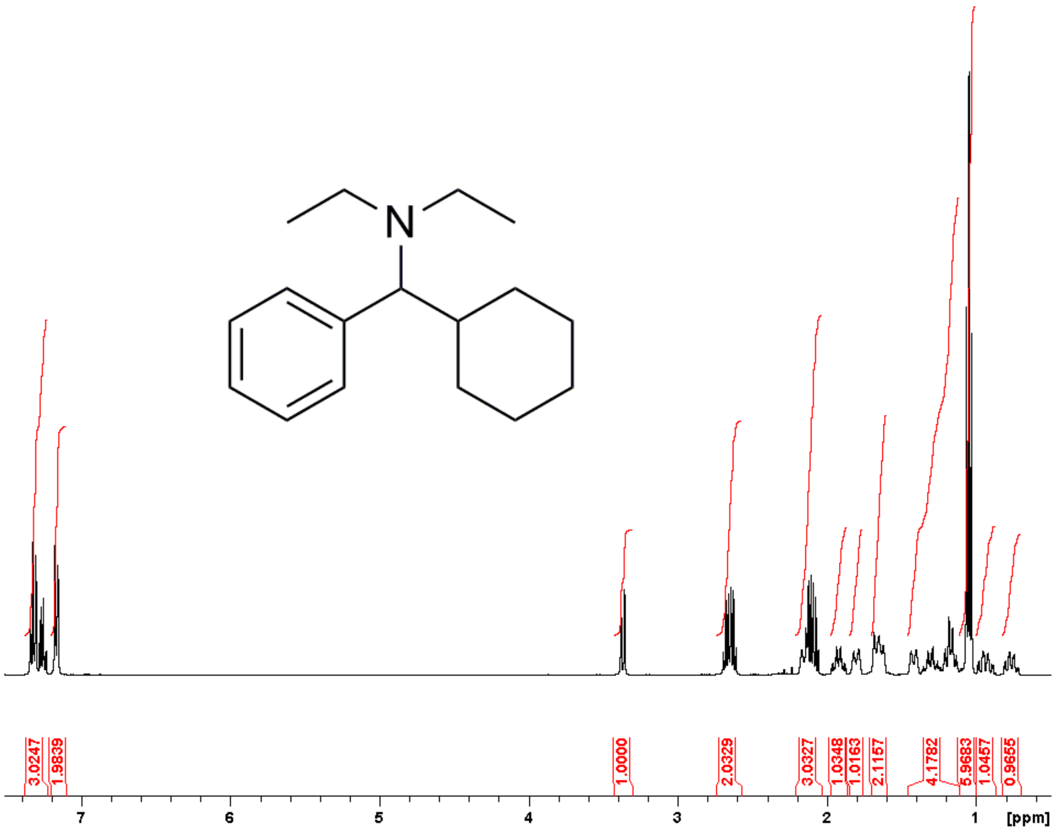
N
3.0247
1.9839
1.0000
2.0329
3.0327
1.0348
1.0163
2.1157
4.1782
5.9683
1.0457
0.9655
7
6
5
4
3
2
1
[ppm]

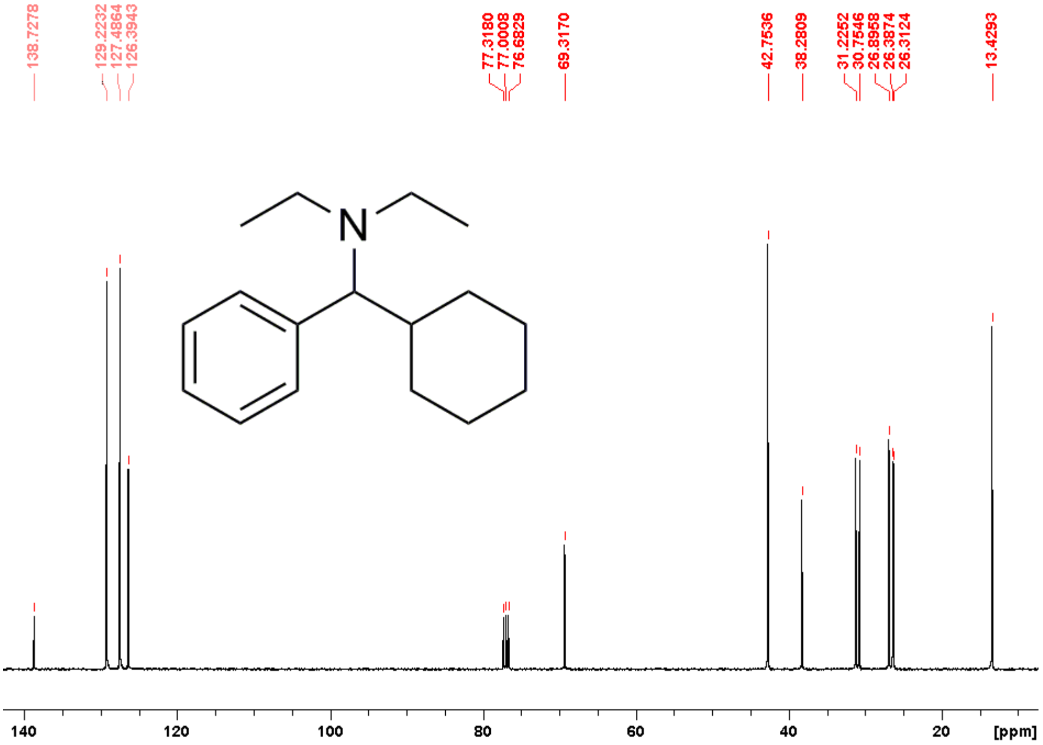
138.7278
129.2232
127.4864
126.3943
77.3180
77.0008
76.6829
69.3170
42.7536
38.2809
31.2252
30.7546
26.8958
26.3874
26.3124
13.4293
N
140 120 100 80 60 40 20 [ppm]

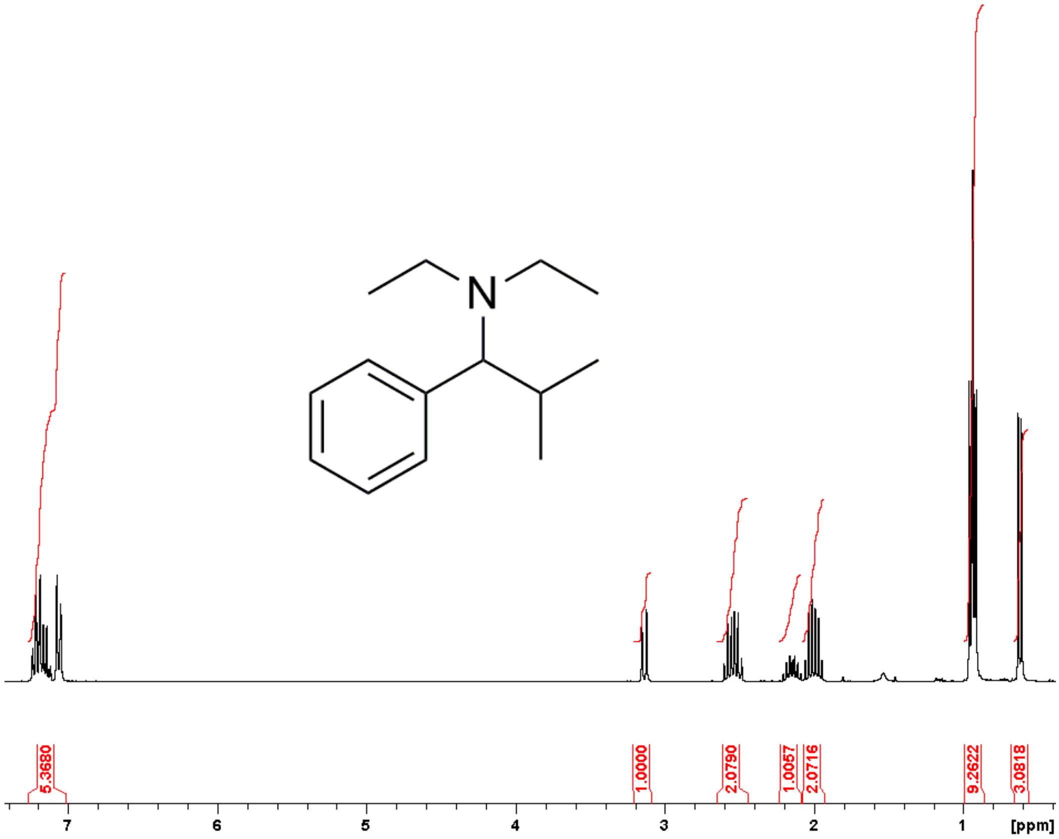
N
5.3680
1.0000
2.0790
1.0057
2.0716
9.2622
3.0818
7 6 5 4 3 2 1 [ppm]

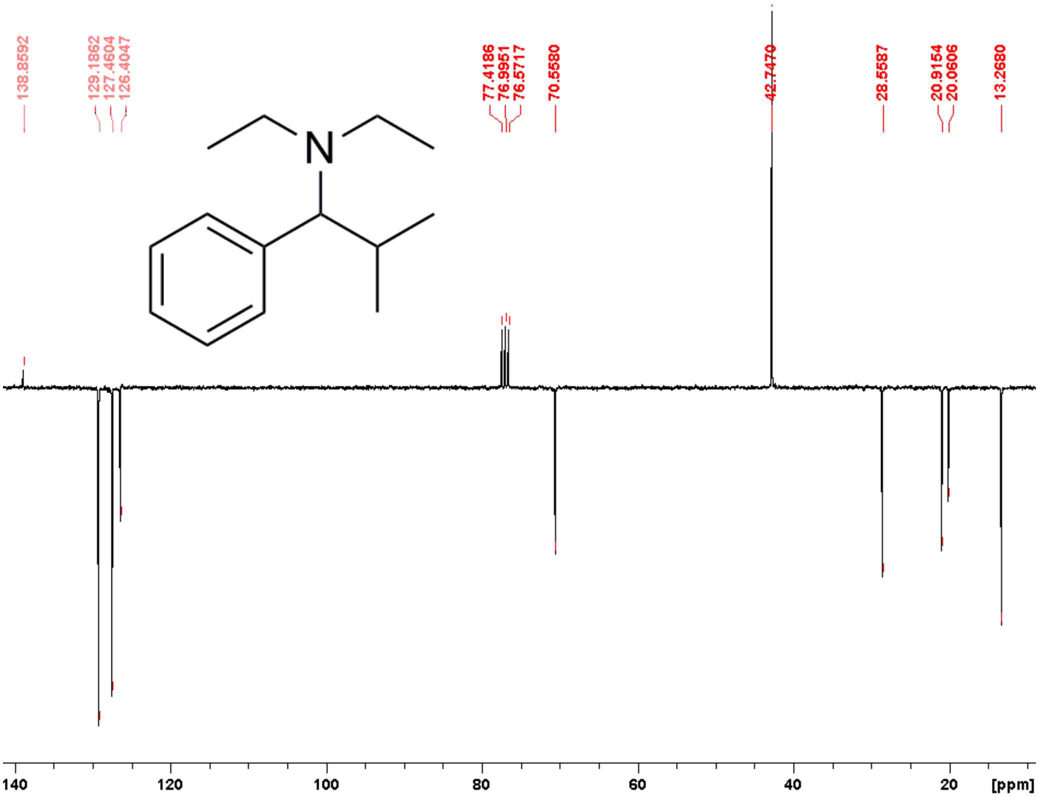
138.8592
129.1862
127.4604
126.4047
77.4186
76.9951
76.5717
70.5580
42.7470
28.5587
20.9154
20.0606
13.2680
N
140
120
100
80
60
40
20
[ppm]

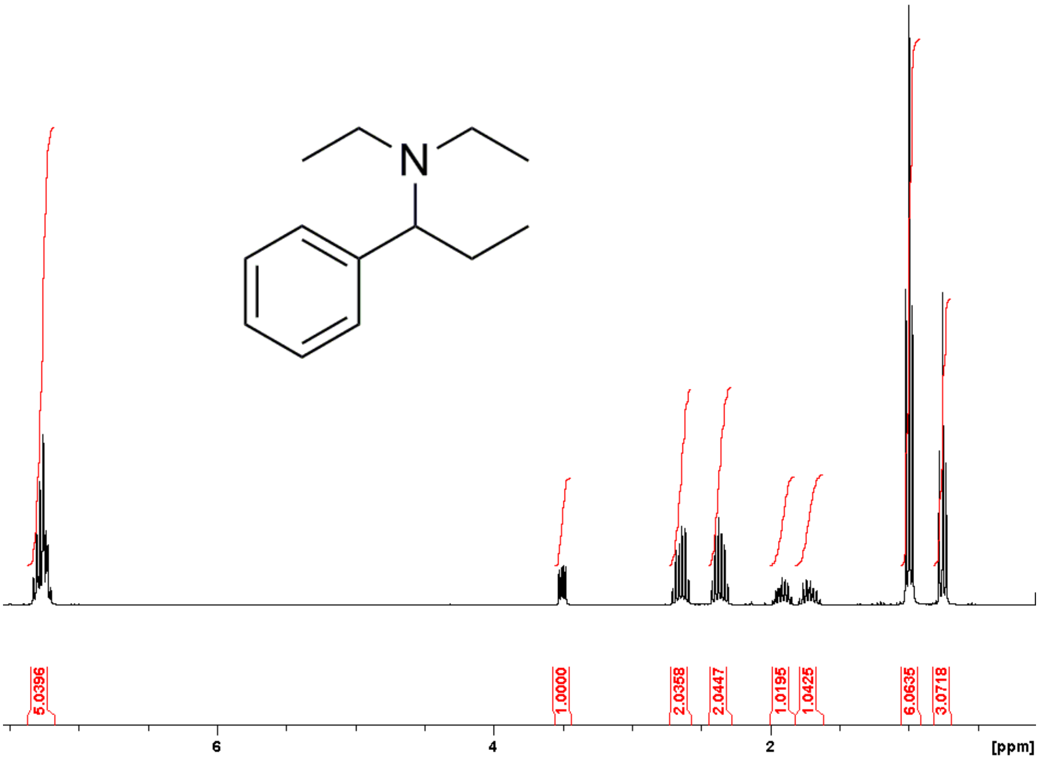
N
5.0396
1.0000
2.0358
2.0447
1.0195
1.0425
6.0635
3.0718
6
4
2
[ppm]

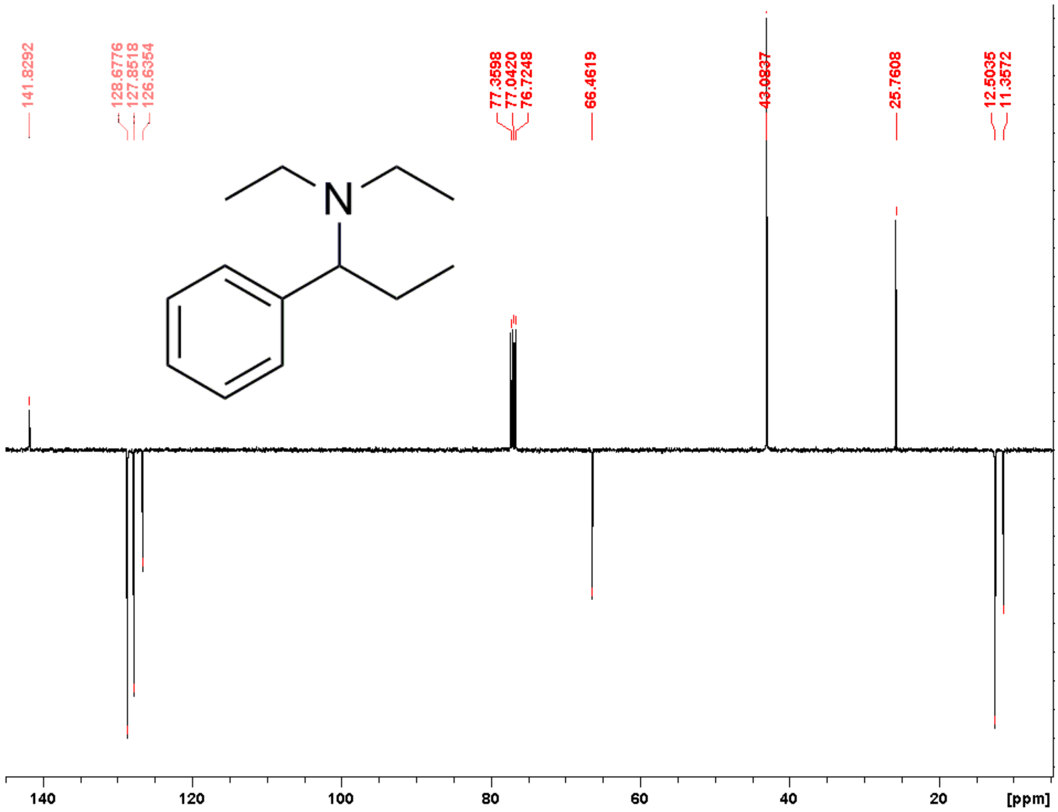
141.8292
128.6776
127.8518
126.6354
77.3598
77.0420
76.7248
66.4619
43.0837
25.7608
12.5035
11.3572
N
140
120
100
80
60
40
20
[ppm]

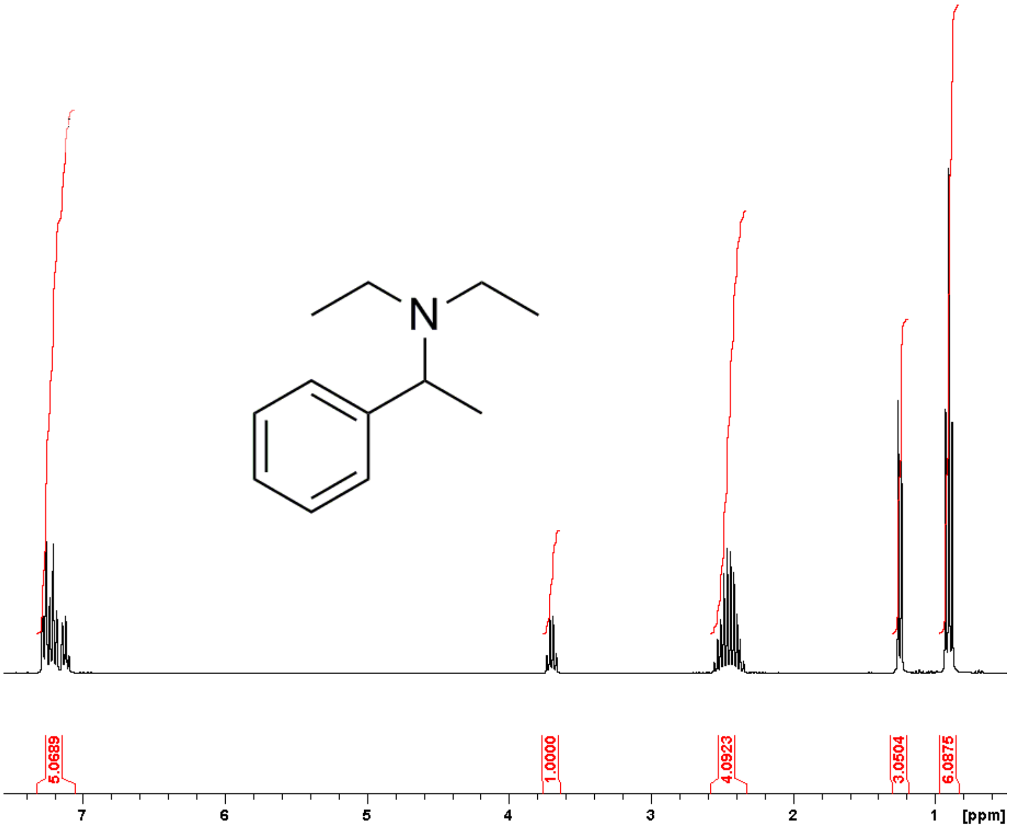
N
5.0689
1.0000
4.0923
3.0504
6.0875
7
6
5
4
3
2
1
[ppm]

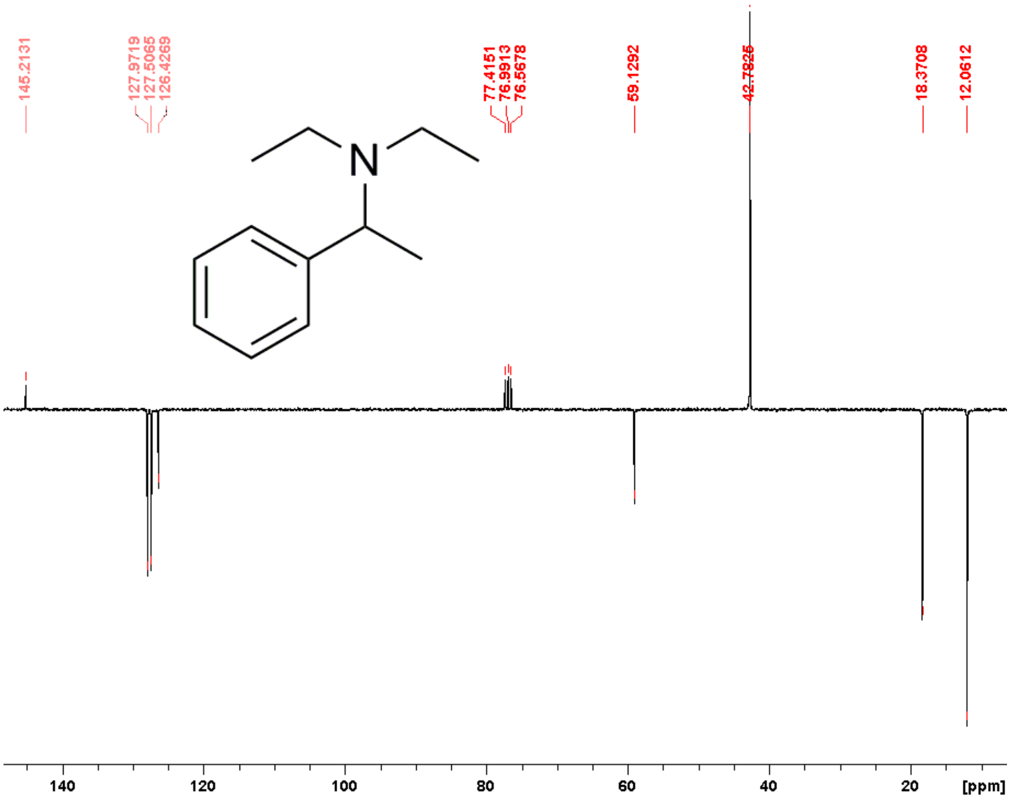
145.2131
127.9719
127.5065
126.4269
77.4151
76.9913
76.5678
59.1292
42.7825
18.3708
12.0612
N
140
120
100
80
60
40
20
[ppm]

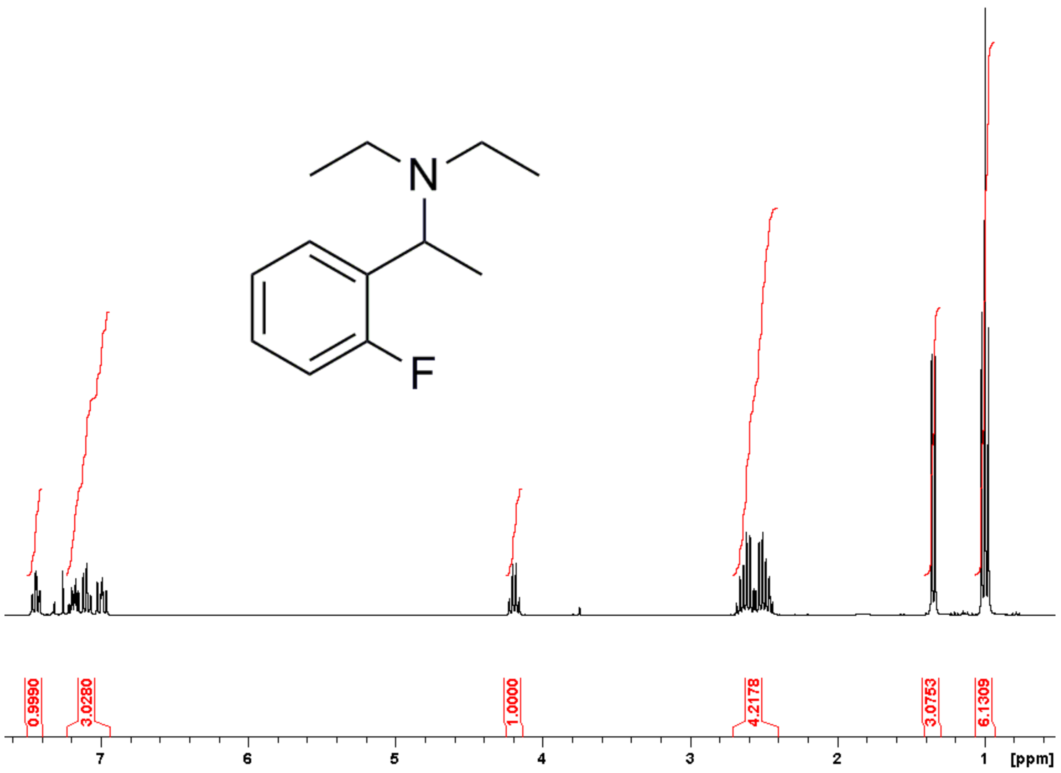
N
F
0.9990
3.0280
1.0000
4.2178
3.0753
6.1309
7
6
5
4
3
2
1
[ppm]

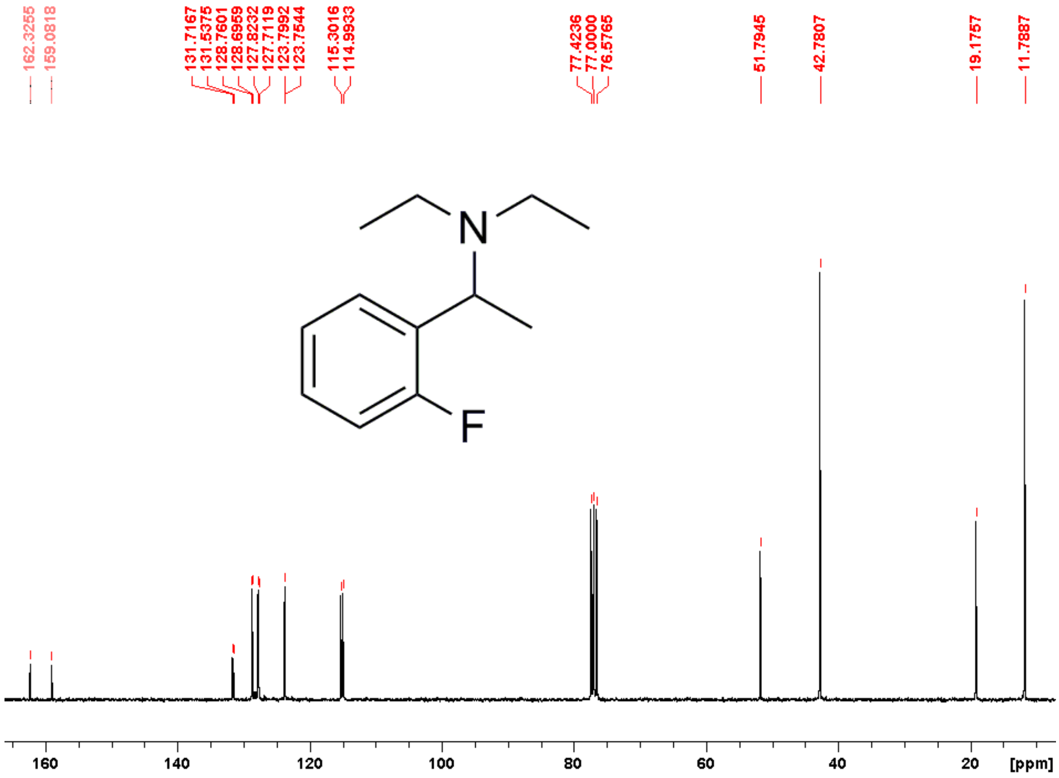
162.3255
159.0818
131.7167
131.5375
128.7601
128.6959
127.8232
127.7119
123.7992
123.7544
115.3016
114.9933
77.4236
77.0000
76.5765
51.7945
42.7807
19.1757
11.7887
N
F
160 140 120 100 80 60 40 20 [ppm]

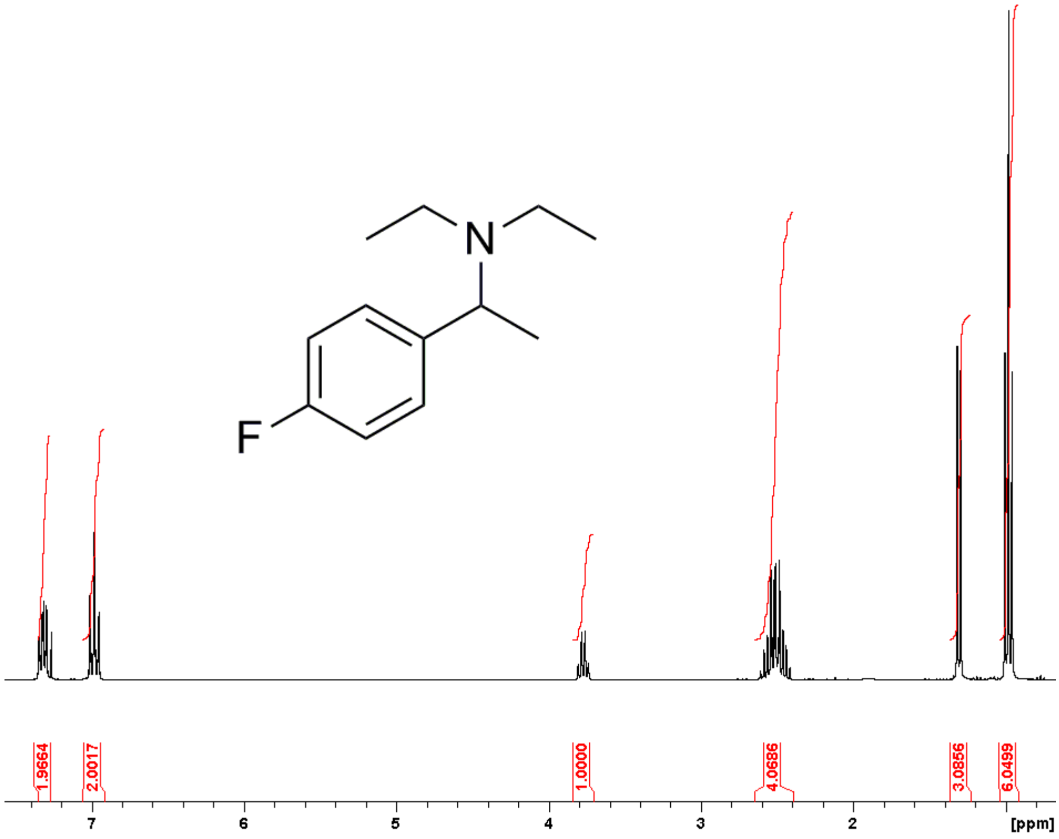
N
F
1.9664
2.0017
1.0000
4.0686
3.0856
6.0499
7 6 5 4 3 2 [ppm]

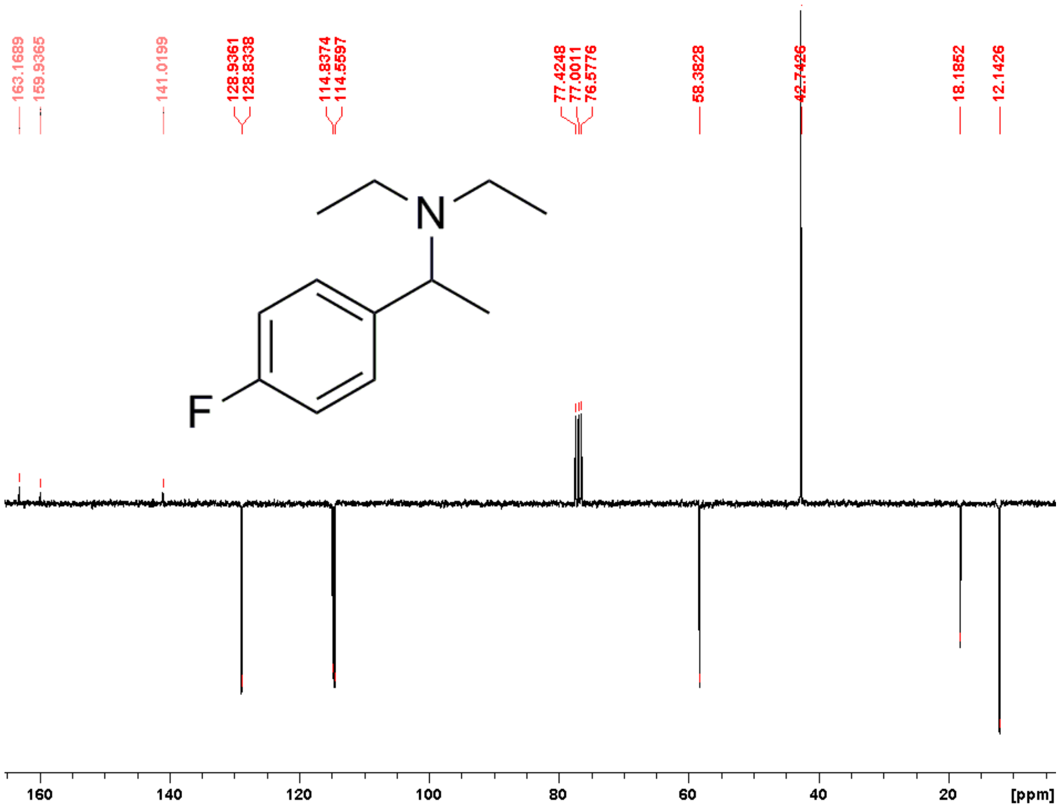
163.1689
159.9365
141.0199
128.9361
128.8338
114.8374
114.5597
77.4248
77.0011
76.5776
58.3828
42.7426
18.1852
12.1426
N
F
160
140
120
100
80
60
40
20
[ppm]

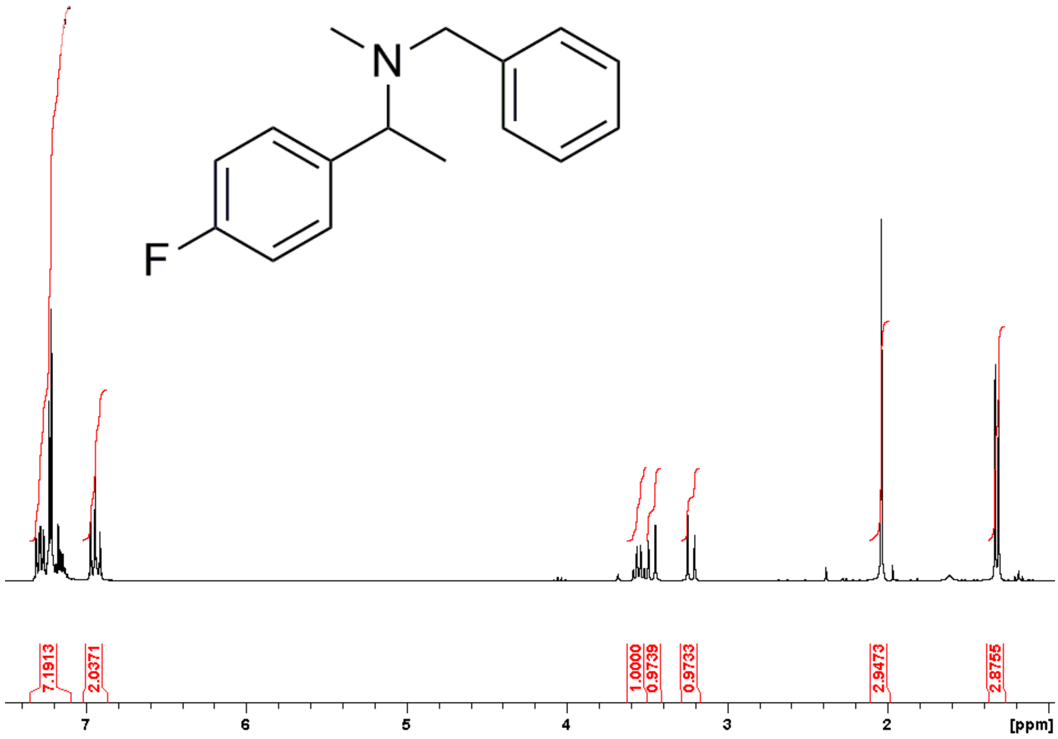
N
F
7.1913
2.0371
1.0000
0.9739
0.9733
2.9473
2.8755
7
6
5
4
3
2
[ppm]

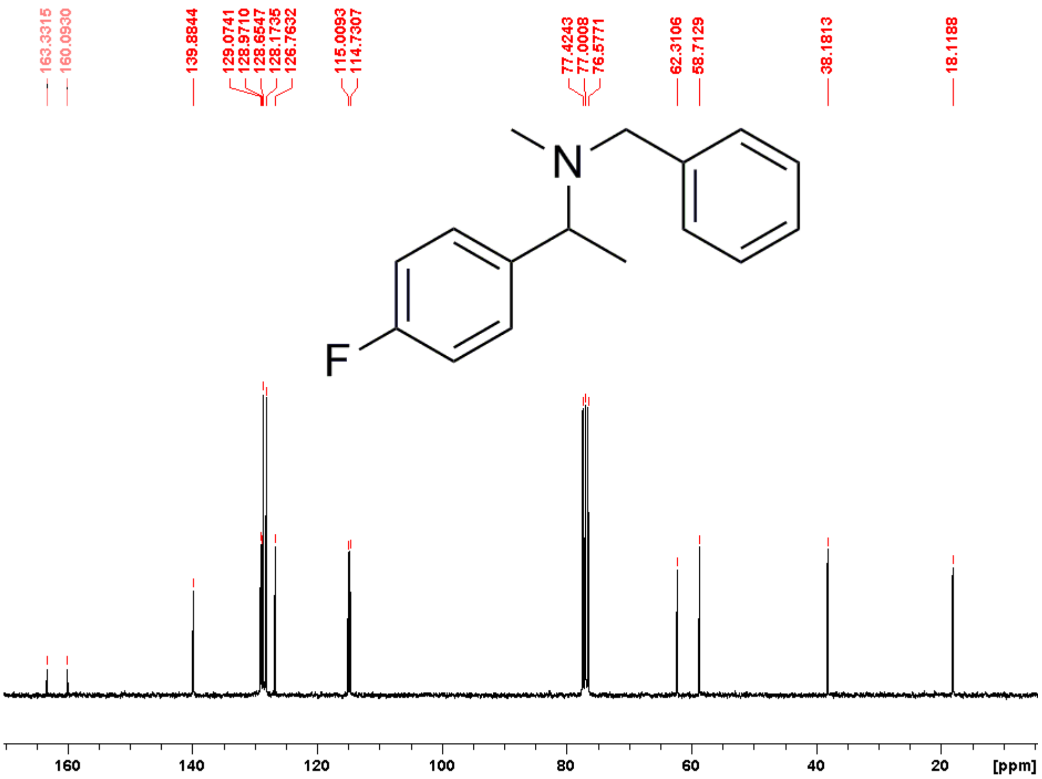
163.3315
160.0930
139.8844
129.0741
128.9710
128.6547
128.1735
126.7632
115.0093
114.7307
77.4243
77.0008
76.5771
62.3106
58.7129
38.1813
18.1188
N
F
160
140
120
100
80
60
40
20
[ppm]

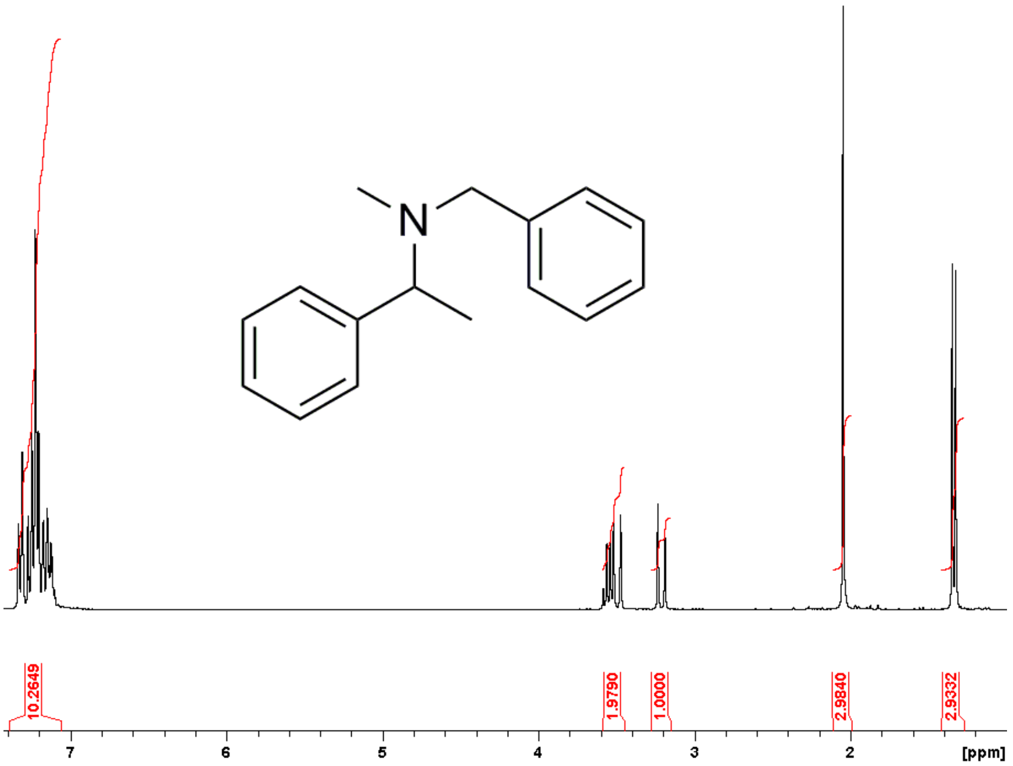
N
10.2649
1.9790
1.0000
2.9840
2.9332
7
6
5
4
3
2
[ppm]

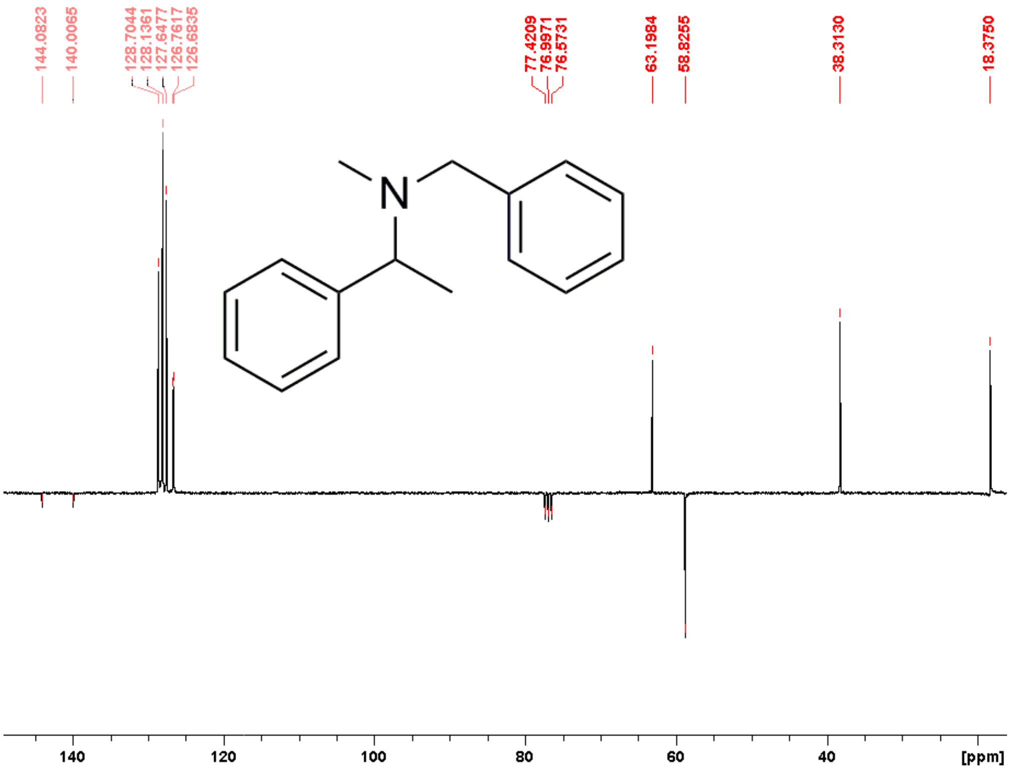
144.0823
140.0065
128.7044
128.1361
127.6477
126.7617
126.6835
77.4209
76.9971
76.5731
63.1984
58.8255
38.3130
18.3750
N
140
120
100
80
60
40
[ppm]

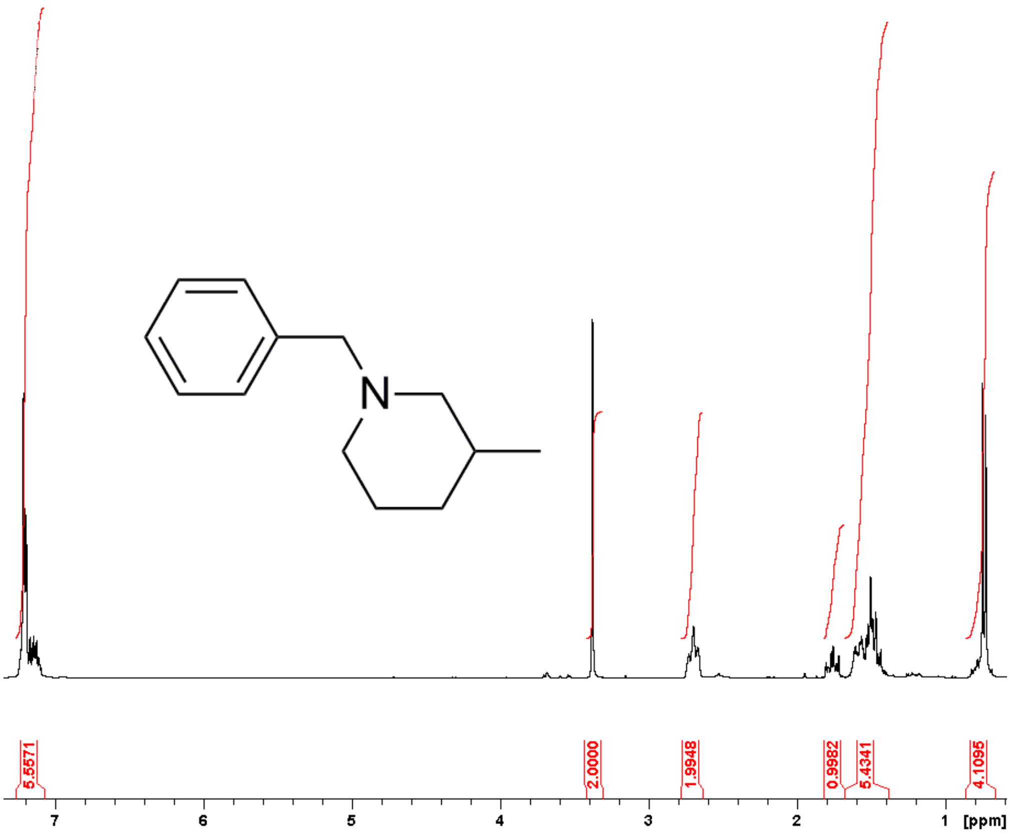
N
5.5571
2.0000
1.9948
0.9982
5.4341
4.1095
7
6
5
4
3
2
1
[ppm]

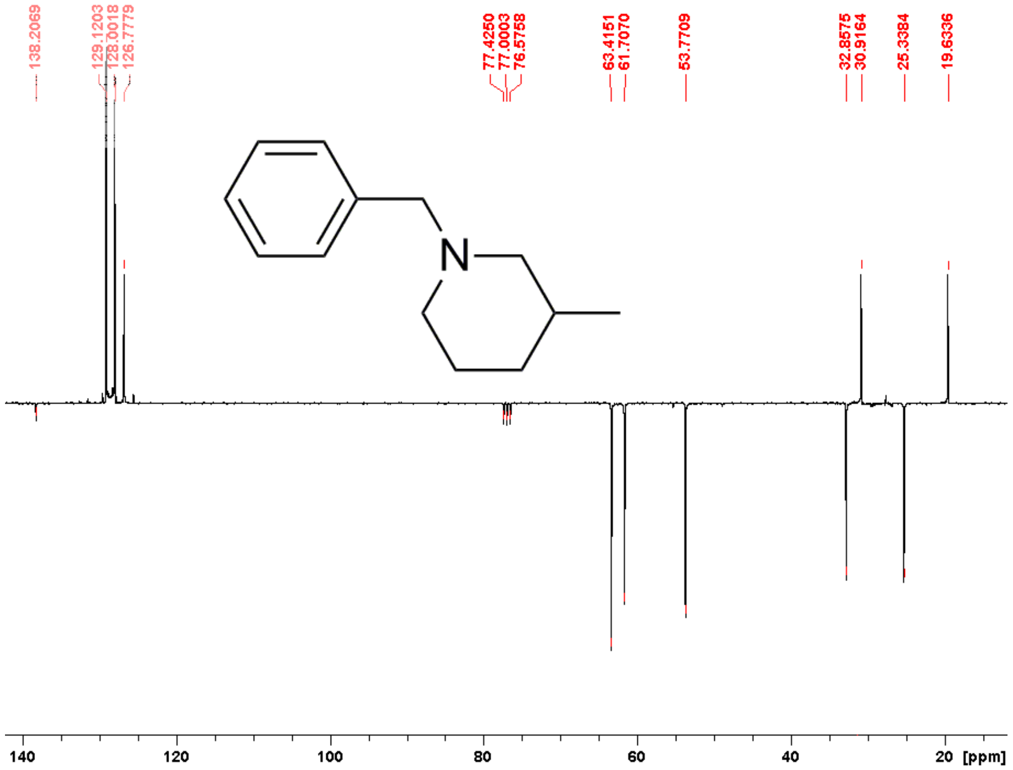

138.2069
129.1203
128.0018
126.7779
77.4250
77.0003
76.5758
63.4151
61.7070
53.7709
32.8575
30.9164
25.3384
19.6336
N
140
120
100
80
60
40
20 [ppm]
